# Untargeted metabolomics and metagenomics reveal signatures for intramammary ceftiofur treatment and lactation stage in the cattle hindgut

**DOI:** 10.3389/fmolb.2024.1364637

**Published:** 2024-05-21

**Authors:** Karla A. Vasco, Zoe A. Hansen, Anthony L. Schilmiller, Bailey Bowcutt, Samantha L. Carbonell, Pamela L. Ruegg, Robert A. Quinn, Lixin Zhang, Shannon D. Manning

**Affiliations:** ^1^ Department of Microbiology, Genetics and Immunology, Michigan State University, East Lansing, MI, United States; ^2^ Research Technology Support Facility, Mass Spectrometry and Metabolomics Core, Michigan State University, East Lansing, MI, United States; ^3^ Department of Large Animal and Clinical Sciences, Michigan State University, East Lansing, MI, United States; ^4^ Department of Biochemistry and Molecular Biology, Michigan State University, East Lansing, MI, United States; ^5^ Department of Epidemiology and Biostatistics, Michigan State University, East Lansing, MI, United States

**Keywords:** metabolomics, gut microbiome, antibiotic use, ceftiofur, metagenomics

## Abstract

The gut microbiota in cattle is essential for protein, energy, and vitamin production and hence, microbiota perturbations can affect cattle performance. This study evaluated the effect of intramammary (IMM) ceftiofur treatment and lactation stage on the functional gut microbiome and metabolome. Forty dairy cows were enrolled at dry-off. Half received IMM ceftiofur and a non-antibiotic teat sealant containing bismuth subnitrate (cases), while the other half received the teat sealant (controls). Fecal samples were collected before treatment at dry off, during the dry period (weeks 1 and 5) and the first week after calving (week 9). Shotgun metagenomic sequencing was applied to predict microbial metabolic pathways whereas untargeted metabolomics was used identify polar and nonpolar metabolites. Compared to controls, long-term changes were observed in the cows given ceftiofur, including a lower abundance of microbial pathways linked to energy production, amino acid biosynthesis, and other vital molecules. The metabolome of treated cows had elevated levels of stachyose, phosphatidylethanolamine diacylglycerol (PE-DAG), and inosine a week after the IMM ceftiofur application, indicating alterations in microbial fermentation, lipid metabolism, energy, and cellular signaling. Differences were also observed by sampling, with cows in late lactation having more diverse metabolic pathways and a unique metabolome containing higher levels of histamine and histamine-producing bacteria. These data illustrate how IMM ceftiofur treatment can alter the functionality of the hindgut metabolome and microbiome. Understanding how antibiotics and lactation stages, which are each characterized by unique diets and physiology, impact the function of resident microbes is critical to define normal gut function in dairy cattle.

## 1 Introduction

The gut microbiota of ruminants produces proteins, vitamins, and ∼75% of the energy necessary for the host through an obligatory symbiotic relationship (Bergman, 1990). Rumen microorganisms ferment the plant biomass to generate energy in the form of volatile fatty acids (VFAs) (Bergman, 1990) and convert nitrogen-containing compounds into protein (Bach et al., 2005). Thus, prior studies have sought to determine the relationship between the microbiome composition of cattle and animal production and methane emissions (reviewed by (O’Hara et al., 2020)). Most notably, microbial communities of the gastrointestinal tract were shown to influence the quality and yield of milk production, affecting key components such as fat, protein, and lactose content (Jami et al., 2014; Xu et al., 2017; Buitenhuis et al., 2019; Wu et al., 2021). Consequently, shifts in the microbiome and metabolome can potentially alter milk composition and affect cow health. Although antibiotics are known to cause perturbations in the gut microbiome, little is known about the specific effects of intramammary (IMM) antibiotic treatment on the function of the fecal microbiome in dairy cows.

β-lactam antibiotics such as ceftiofur, a third-generation cephalosporin, are often used in dairy cattle for the treatment of mastitis or dry cow therapy (Hallberg et al., 2006; Campos et al., 2021). When cephalosporins are applied intramammarily, they are mainly excreted through the urine and udder (Wilson and Gilbert, 1986; Rule et al., 1998; Ray et al., 2014). Yet, ∼13% of the IMM-administered ceftiofur dose in lactating cows, which includes two doses of 125 mg per quarter given 12 h apart, is detectable in the feces 5–6 days post-treatment (European Agency for the Evaluation of Medicinal Products, 2002). When administered subcutaneously to Holstein steers, ceftiofur active metabolites were shown to alter the microbiota composition of the gut (Foster et al., 2019) due to activity against both Gram-negative and Gram-positive bacteria. Our previous study of Holstein cows given IMM ceftiofur treatment at dry-off also showed an altered abundance of specific taxa in the short and long-term, although no effect was observed on microbiota diversity (Vasco et al., 2023). Specifically, we observed a higher abundance of Actinobacteria and Bacteroidetes and lower abundance of Proteobacteria and Firmicutes in the cows given IMM antibiotics at dry off *versus* untreated cattle over a 9-week period. It is therefore possible that these taxa play an important role in the function of the gut microbiota during antibiotic therapy.

To examine the function of microbial communities, metagenomic approaches have been applied that enable the prediction of microbial metabolic capacity based on the detection of genes encoding enzymes and mapping them onto metabolic pathways (Beghini et al., 2021). The characterization of metabolites from host, dietary, and microbiome sources can also provide a better understanding of the functional interactions between the microbiome and environment. Untargeted metabolomics, for instance, uses liquid chromatography–tandem mass spectrometry (LC–MS/MS) to simultaneously detect multiple compounds based on their retention time and spectral fragmentation patterns (MS/MS) (Rakusanova et al., 2023). Metabolomics of the rumen content of dairy cows has improved understanding of diet-related metabolism while defining how it is influenced by the introduction of grain into the diet (Saleem et al., 2012) and identifying differences between fecal and rumen metabolites (Malheiros et al., 2021). Furthermore, integrated ‘omics approaches such as metagenomics, metatranscriptomics, and metabolomics, have been used to characterize the functional microbiome in the rumen to identify microbial features linked to feed efficiency (Xue et al., 2022).

Since we demonstrated that IMM ceftiofur treatment of dairy cattle impacted the fecal microbiota and antibiotic-resistant bacterial populations when compared to cows without treatment (Vasco et al., 2023), we sought to characterize the function of the hindgut microbiome and metabolome in the same dairy cows. To identify short- and long-term changes due to antibiotic therapy, samples were taken a day prior to dry-off and ceftiofur treatment and again at 1 and 9 weeks later as described (Vasco et al., 2023). These time points correspond to three different stages of lactation and include late lactation (day -1), dry-off (week 1), and the periparturient period (week 9).

The different stages of lactation differ with respect to the diet given to the cows but also their physiology as outlined by the National Research Council (National Research Council, 2001). Indeed, cows in late lactation require a maintenance diet containing high levels of metabolizable protein and energy. During the dry period when cows are not producing milk, however, the mammary gland and udder tissue will involute and regenerate before the next lactation. The dry period lasts about 60 days prior to calving. As opposed to lactation, cows require lower quantities of metabolizable energy in their diet during the dry period. Comparatively, early lactation lasts approximately 30 days post-calving and represents the start of the lactation period. Higher levels of energy, calcium, and metabolizable protein are required for fresh cows when compared to dry cows to compensate for the energy imbalance induced by milk production and low dry-matter intake (National Research Council, 2001). This energy deficit generally persists through the 60th day of lactation, after which the cows shift to a net positive energy state. Since dietary changes are also linked to alterations in the gut microbiota in dairy cattle (Lin et al., 2023), we applied multi-omics approaches to identify interactions between the microbiome and metabolites present in fecal samples from ceftiofur-treated and ceftiofur-untreated dairy cows during different stages of lactation. The findings of this study enhance understanding of the effects that both ceftiofur treatment and lactation stage have on the function of the gut microbiome.

## 2 Materials and methods

### 2.1 Study population and epidemiological data

Forty Holstein cows were enrolled at the start of the dry-off period in June-November of 2019 at the Michigan State University (MSU) Dairy Cattle Teaching and Research Center as described (Vasco et al., 2023). After the last milking, twenty cows (cases) received a single IMM infusion containing 500 mg of ceftiofur hydrochloride (CHCL; SpectramastDC^®^; Zoetis Animal Health) along with a non-antibiotic teat sealant with bismuth subnitrate (Orbeseal^®^; Zoetis Animal Health) in each teat (*n* = 4; total of 2 g of CHCL). Cows in the control group (*n =* 20) received only the IMM teat sealant. All cows had a somatic cell count (SCC) of <150,000 cells/mL at the most recent Dairy Herd Improvement Association test and none received antibiotics in the prior 90 days of lactation. The study protocol was approved by the Institutional Animal Care and Use Committee at MSU (IACUC number ROTO201800166) prior to sampling.

To avoid a parity effect, controls were matched to the ceftiofur-treated cows based on parity as well as monthly milk production. Diet regimens were formulated using Spartan Dairy 3™ software per guidelines outlined in the Nutrient Requirements of Dairy Cattle report (National Research Council, 2001). Based on the dietary information extracted from farm records, different diets were given to the cows in accordance with their production demands across the sampling period (Table 1); the matched treated and control cows were given the same diets within each lactation stage. Near the end of lactation, which corresponded to a day prior to the IMM treatment (day -1), cows received the maintenance diet containing 14% more metabolizable energy and 2.5 times more metabolizable protein (g) than is provided in the dry-off diet (weeks 1 through 5) (Supplementary Table S1). At week 9, animals were given a diet for fresh cows consisting of 64% of dry matter intake when compared to lactating cows, but with transitioning levels of energy and protein that were 15% and 64% higher than during dry-off, respectively.

**TABLE 1:** Diet rations fed to dairy cows at four different lactation stages.

Ration component	Maintenance	Early dry	Close-up	Fresh
As-Fed (kg)	48.77	32.58	19.89	31.35
DM Fed (kg)	24.04	12.98	12.74	15.89
Corn grain ground fine (DM fed kg)	4.31	0	0	2.27
Corn gluten feed dry (DM fed kg)	1.81	0	0	0.45
Soybean Hulls Pellet (DM fed kg)	2.04	0	0	0
Soybean meal 475 solvent (DM fed kg)	1.13	0.95	2.72	1.36
Cottonseed Fuzzy (DM fed kg)	1.36	0	0	0
MSU Corn silage (DM fed kg)	4.08	4.08	3.8	5.58
MSU Haylage (DM fed kg)	4.08	4.04	0	2.72
CFE MSU dairy base (DM fed kg)	0.45	0.27	0.32	0.36
MSU Long bunk BMR CS (DM fed kg)	3.63	0	0	0
MSU fresh high supplement (DM fed kg)	1.13	0	0	1.07
MSU Purchased Alfalfa Hay (DM fed kg)	0	0	0	2.09
CFE MSU PreFresh DE (DM fed kg)	0	0	0.45	0
MSU Low K Grass Hay (DM fed kg)	0	0	4.76	0
SoyChlor (DM fed kg)	0	0	0.69	0
MSU grasslage (DM fed kg)	0	2.72	0	0
MSU Straw (DM fed kg)	0	0.91	0	0
Grass Pasture 16 CP 55 NDF 7 LNDF (DM fed kg)	0	0	0	0
QLF Ignite Dry Cow 25 (tub) (DM fed kg)	0	0	0	0

DM, dry matter; NDF, neutral detergent fiber; LNDF, Lignin as a percent of the NDF; CP, crude protein; CS, corn silage; BMR, brown midrib.

Animals in all phases received corn silage, soybean meal with 47.5% crude protein, CFE MSU dairy base, and haylage. It was only during the lactation and fresh periods that the ration included corn (ground fine and fed dry) as well as MSU fresh high supplement to increase the energy density and provide essential nutrients such as calcium, magnesium, potassium, and niacin to prevent metabolic disorders that can occur during the transition into lactation. Comparatively, soybean hull pellets, cottonseed, and long bulk brown midrib (BMR) corn silage (CS) were exclusively given to cows in late lactation, while alfalfa hay was only provided to fresh cows. Although grass silage, MSU straw, grass pasture, and ignite supplement (Quality Liquid Feeds^®^, WI, Unites States) were given to all animals, they were only provided during the dry period in small quantities. The ignite supplement contains 25% protein, fat, trace minerals, and vitamins A, D, and E (https://onealsfarmandgarden.com/products/tub-qlf-ignite-30).

### 2.2 Sample collection and processing

**FIGURE 1:**
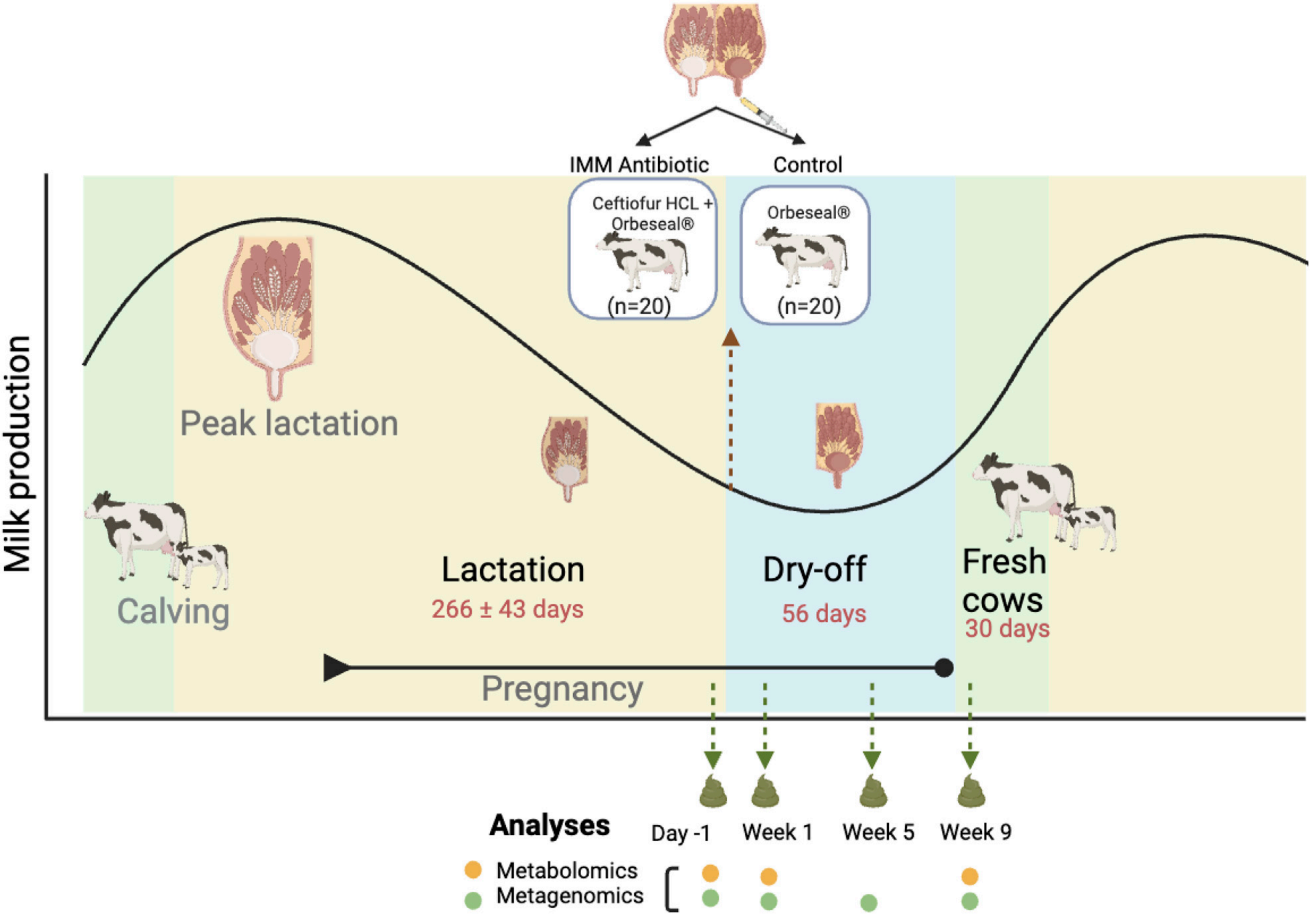
Diagram illustrating the animal treatment schedule and sample collections across the stages of lactation. The fecal sampling regimen coincided with three key lactation stages. These include: 1) the end of lactation (1 day before the initiation of dry cow therapy with intramammary (IMM) ceftiofur, or Day -1); 2) after dry cow therapy during the dry-off period (Weeks 1 and 5); and 3) 9 weeks after treatment at the end of the dry-off period and beginning of the fresh phase (Week 9). The black line demonstrates the fluctuations in milk production across each stage.

Fecal samples were collected from all cows at four time points corresponding to the different stages of lactation (Figure 1). These stages included late lactation (day -1), dry-off (weeks 1 and 5), and the periparturient period (week 9). Along with physiological differences, the dietary needs of the animals and feed formulations differ across the three lactation stages. Samples were collected before ceftiofur administration at the end of lactation when the cows received a maintenance diet (day -1) as well as after dry-off (week 1; week 5) when they were given an early dry diet. The final sample was collected during or just prior to calving (week 9) when the cows were given the fresh formulation. One cow could not be sampled at week 9 due to birthing by cesarean section that required antibiotics treatment, leaving 79 samples for analysis from the 9-week sampling point and 159 samples in all. As indicated previously (Vasco et al., 2023), the fecal samples were collected via the rectum using clean obstetric sleeves and transported in a cooler to MSU in sterilized sampling bags for processing.

Each sample was homogenized by hand and aliquots containing 0.25 g of feces and 0.25 g of feces in 750 µL of 190 Proof ethanol were stored for metabolite and DNA extractions, respectively. All fecal aliquots were flash frozen with liquid nitrogen for 1 min and were stored at −80°C until further processing. The 119 samples collected at the day -1, week 1, and week 9 samplings were used for both untargeted metabolomics and metagenomic sequencing, while metagenomic sequencing data were also available for the 40 samples collected at week 5 through our prior study (Vasco et al., 2023). Since we previously observed similar microbiota diversity and composition in the samples collected at weeks 1 and 5, metabolomics was not applied to the week 5 (dry-off) samples.

### 2.3 Metagenomics analyses

#### 2.3.1 Metagenomic sequencing

Fecal samples were centrifuged for 5 min at 16,000 rpm at 4°C to remove the supernatant and the pellets were washed twice with 1 mL of 1× PBS as described (Vasco et al., 2023). DNA was extracted with the DNeasy PowerSoil Pro Kit (Qiagen, Germantown, MD, United States) using the manufacturer’s protocol followed by an additional wash step using the C5 solution to improve quality. Samples with an average dsDNA concentration of 1,277.3 ng (±310.5 ng) as measured using a Qubit, were sent to CosmosID (Rockville, MD, United States) for metagenomic next-generation sequencing (mNGS). The Nextera™ XT DNA Library Preparation Kit (Illumina, San Diego, CA, United States) was used on all samples and sequencing was performed using the Illumina HiSeq X platform (2 × 150 bp).

#### 2.3.2 Microbiome characterization

Metagenomic analyses to characterize the gut microbiota and resistome in the same cows were described previously (Vasco et al., 2023). Briefly, removal of bovine DNA and adapter sequences was performed and the microbiome and resistome composition were analyzed with MetaPhlan4 (Blanco-Miguez et al., 2023) and Resistome Gene Identifier (Alcock et al., 2020), respectively. The software KMA was used to identify plasmids, virulence genes and viruses using the databases PLSDB (Schmartz et al., 2022), VFDB (Chen et al., 2005), and Virus-Host (Mihara et al., 2016). Abundance scores were determined based on genome equivalents and the number of reads to calculate the relative abundance of taxa and genes.

An evaluation of the function of the cattle microbiome was performed using the HUMAnN 3.0 pipeline (Beghini et al., 2021), which allows for the identification of metabolic pathways with their microbial species-level contributions. The following databases were used: ChocoPhlAn 3 (Beghini et al., 2021) for taxonomic identification, UniRef90 (Steinegger and Söding, 2018) for enzyme commission number screening, and MetaCyc v24.0 (Caspi et al., 2020) for the assignation of pathways. First, paired-raw sequences were processed with Trimommatic v.0.39 (Bolger et al., 2014) to remove low-quality reads and adapters used for Illumina sequencing. Burrows-Wheeler Aligner v.0.7.15 (Li, 2013) and SAMtools v.1.4.1 (Danecek et al., 2021) removed bovine DNA reads [*Bos taurus*, ARS-UCD1.2 (Rosen et al., 2020)]. Trimmed non-host paired FASTQ reads were merged with the UNIX command ‘cat’. Merged reads were used as input for HUMAnN 3.0 and the resulting pathway abundances, reported as reads-per-kilobase (RPK), were normalized as the relative abundance per sample. A joined matrix containing the pathway relative abundances for all samples was generated with the command “humann_join_tables”, whereas pathways of interest were depicted with the “humann_barplot” function while stratifying the pathway contributions by bacterial taxa (https://github.com/biobakery/humann).

### 2.4 Extraction of metabolites from cattle feces

Metabolite extractions were performed on all 119 fecal samples collected at day -1 (*n* = 40), week 1 (*n* = 40), and week 9 (*n* = 39). Internal standard solutions were prepared for quality control and normalization including: 1) labeled short-chain fatty acids (SCFAs) (10 µM each of [^13^C]sodium formate, [^13^C_2_]sodium acetate, [^13^C_3_]sodium propionate, and [^13^C_4_]sodium butyrate) in 50:50 (v/v) methanol/water; 2)) [^13^C_16_]palmitic acid (10 µM in 100% isopropanol); 3) phenylalanine-d_7_ (10 µM in 50:50 methanol/water); 4) succinic acid-d_4_ (10 µM in 50:50 methanol/water); and 5) labeled bile acids (10 µM each of glycocholic acid-*d*_4_ and glycoursodeoxycholic acid-*d*_4_ in 50:50 methanol/water). A total of 20 mg of feces was weighed under sterile conditions and 350 µL of ice-cold methanol containing 0.1% butylated hydroxytoluene (BHT) was added. The sample was homogenized and incubated on ice for 10 min. For feces sedimentation, 10 µL of each standard was mixed into the samples, agitated for 30 s, and centrifuged at 10,000 × rpm at 4°C for 10 min. The supernatant was pipette-transferred to a sterile microcentrifuge tube on ice, while ice-cold HPLC-grade isopropanol (200 µL) was added to the pellet, homogenized for 30 s, and centrifuged at 10,000 × rpm and 4°C for 10 min. Finally, the isopropanol supernatant was combined with the initial extract and 100 µL aliquots of the mixed extracts were stored into glass vials inserted in 2-mL amber glass autosampler vials sealed with 9 mm screw septum caps. Metabolite extracts were preserved at −80°C until analyzed.

### 2.5 Metabolomics analyses

#### 2.5.1 Untargeted metabolomics

Polar and nonpolar positive metabolites, which are a group of metabolites that carry a net positive charge, were analyzed through LC-MS/MS in a Thermo Scientific Vanquish™ Ultra High-Performance Liquid Chromatography (UHPLC) coupled to a Q Exactive™ Hybrid Quadrupole-Orbitrap™ mass spectrometer (MS). Metabolites with a net negative charge were not evaluated in this study. Along with the samples (*n =* 119), three blanks and pools were included at the beginning of each run (polar and nonpolar) and for every 20 samples. The Xcalibur™ software (ThermoFisher Scientific™, United States) was used for method setup and data acquisition.

The analysis of polar and nonpolar metabolites was conducted using distinct chromatographic conditions tailored to the properties of each metabolite class. Nonpolar metabolites were detected with reversed-phase chromatography using 10 µL of each sample injected with a column Waters Acquity Ethylene Bridged Hybrid (BEH)-C18 UPLC (2.1 × 100 mm) at 60°C. A 0.4 mL/min flow rate was used for a gradient analysis that consisted of 98% mobile phase A (water plus 0.1% formic acid) and 2% mobile phase B (acetonitrile plus 0.1% formic acid) for 1 min. Mobile phase B was ramped to 100% at minute 8 and was held for 2 min. Lastly, mobile phase B was returned to 2% at 10.01 min and held at that concentration for two more minutes.

By contrast, polar metabolites were detected through hydrophilic interaction liquid chromatography (HILIC). A Waters BEH-Amide UPLC column (2.1 × 100 mm) held at 60°C was used to inject 10 µL of sample. The gradient analysis was carried out at a rate of 0.4 mL/min starting with 100% mobile phase B (10 mM ammonium formate/10 mM ammonium hydroxide in 95:5 acetonitrile/water (v/v)) and 0% mobile phase A (10 mM ammonium formate/10 mM ammonium hydroxide in water) for 1 min. Mobile phase B was ramped to 40% at minute 8 and held at this concentration for 2 min. Mobile phase B was returned to 100% at minute 10.01 and held at this concentration for 2 min.

Data were acquired using a data-dependent MS/MS method with electrospray ionization in positive mode and capillary voltage of 3.5 kV, transfer capillary temperature at 262.5°C, sheath gas at 50, auxiliary gas at 12.5, probe heater at 425°C, and S-lens RF level at 50. Survey scans were acquired at 35,000 resolution, automatic gain control (AGC) target of 1E6, maximum inject time 100 m, and m/z range 100–1,500. The top 5 ions were selected for MS/MS with a resolution setting of 17,500, AGC target of 1E5, minimum AGC of 5E3, maximum inject time 50 m, isolation window of 1.5, fixed first mass at m/z 50, dynamic exclusion setting of 3 s and stepped normalized collision energy settings of 20, 40 and 60.

#### 2.5.2 Mass-spectrometry (MS) data processing

Raw files (.RAW) for each sample were transformed to mzXML format with the Global Natural Product Social Molecular Networking (GNPS) conversion software. MS data processing was performed using MZmine v2.53 (Pluskal et al., 2010) while analyzing the polar and nonpolar files separately. Instead of using standards for comparison, we determined the noise levels for MS1 and MS2 (centroided spectrum type) using the blanks and pools. First, mzXML files were imported to MZmine for mass detection at the levels MS1 and MS2 using a noise level of 4E04 for MS1 and 3.5E03 for MS2, which was set based on visual analyses of chromatograms from the pools and blanks. Chromatograms were built with the ADAP (Automated Data Analysis Pipeline) (Myers et al., 2017) module using a scan retention time of 1.00–10.00 min for MS level 1, minimum group size in number of scans equal to 4, group intensity threshold of 4.0E4, minimum highest intensity of 5.0E4, and scan to scan accuracy of 0.002 m/z or 10.00 ppm.

Chromatograms were smoothed using the Savitzky Golay algorithm with a filter width of 5 and deconvoluted with local minimum feature resolver. The deconvolution settings included MS/MS scan pairing with a retention time tolerance of 0.15 absolute min and MS1 to MS2 precursor tolerance of 0.002 Da. Additionally, the deconvolution algorithm was set up with a chromatographic threshold of 83.3999%, minimum search range RT/Mobility (absolute) of 0.05, minimum relative height of 0.0%, minimum absolute height of 5.0E4, min ratio of peak top/edge 1.80, and peak duration range (min/mobility) 0.00–1.51. Isotopes were grouped with a m/z tolerance of 0.0015 m/z or 3.0 ppm, a retention time tolerance of 0.05 absolute mins, and a maximum charge of 2 while choosing the most intense representative isotopes.

Next, an aligned feature list containing data from all samples was generated with module join aligner using a tolerance of 0.0015 m/z or 5.0 ppm, weight for m/z of 3, retention time (RT) tolerance of 0.1 absolute min, and weight for RT of 1. Gaps in the aligned list were filled with the module peak finder using an intensity tolerance of 20%, an m/z tolerance of 0.002 m/z or 10.0 ppm, and a retention time tolerance of 0.05 absolute min. Duplicate peaks generated during gap filling were removed at a m/z tolerance of 8.0E-4 m/z or 1.5 ppm and an RT tolerance of 0.035 absolute (min). To identify only those features present in at least three samples, the module “feature list rows filter” was used with at least 3 peaks in a row, keeping only peaks with MS2 scan, and resetting the peak number ID. Finally, the feature list was exported for analyses in GNPS for the Feature-Based Molecular Networking (FBMN) workflow using filter rows only with MS2. The exported files consisted of a feature quantification table (.CSV format) and an MS/MS spectral summary file (.MGF format) with a list of MS/MS spectra associated with the LC-MS/MS ion features.

#### 2.5.3 Metabolite classification

The FBMN workflow in GNPS was used (Wang et al., 2016; Nothias et al., 2020) after importing the MGF file and feature quantification table generated in MZmine as well as the metadata containing the sample attributes. Precursor ion mass and fragment ion mass tolerances were set at 0.02 Da. Default settings were used for the advanced options except for minimum matched fragment ions for networks and library search min matched peaks, which were set at 4. All the spectra with IDs were downloaded; library ID and the network component index were recorded for each metabolite and are referred to as “cluster” for the downstream analyses. Molecular networks were visualized in GNPS to identify metabolite components and clusters of interest.

#### 2.5.4 Metabolome data analyses

The R package Phyloseq v.1.38 was used to analyze metabolomics diversity and composition (McMurdie and Holmes, 2013). A Phyloseq object was generated by merging metadata with the feature table containing cluster intensities, and the cluster identifications, which included three levels: network component, library ID and cluster numbers. The R package decontam v.1.14 (Davis et al., 2018) was used to remove contaminant clusters associated with the standards based on a combined method that uses the Fisher’s exact test. This method concatenates the probabilities of a cluster being present in a sample based on the amount of feces used for the metabolite extraction and the prevalence of a given cluster in controls versus the samples. Although the standards were present in the blanks and assessed separately, they were excluded from the final analysis. Lastly, cluster intensities were normalized to their relative abundances per sample.

#### 2.5.5 Metabolome diversity analyses

The alpha diversity was calculated using the Shannon index and the number of observed features. The paired, one-tailed Wilcoxon signed-rank test was used to compare alpha diversity between groups and time points, whereas the Friedman’s test was used to compare the indexes by animal over time since it accounts for repeated measures. Differences in beta-diversity or metabolome composition, were evaluated based on Bray-Curtis dissimilarity distances that were mapped with Principal Coordinate Analyses (PCoA) using the R packages Vegan v.2.5-7 (Dixon, 2003) and ggplot2 v.3.3.5 (Wickham, 2011). The mean compositions, represented by the centroid of each group of samples in the PCoA, were compared with permutational multivariate analysis of variance (PERMANOVA) with 999 permutations, while dispersion was compared with PERMDISP (Anderson, 2006).

### 2.6 Statistical analyses

#### 2.6.1 Detecting significantly different features between groups

To detect significantly different features between the treatment groups and stages of lactation (time points), the following were used: 1) Linear Discriminant Analysis (LDA) Effect Size (LEfSe) (Segata et al., 2011); 2) Analysis of compositions of microbiomes with bias correction (ANCOM-BC) (Lin and Peddada, 2020); and 3) Microbiome Multivariable Associations with Linear Models (MaAsLin2) (Mallick et al., 2021), as suggested in a prior study (Nearing et al., 2022). LEfSe analysis was performed on normalized log2 abundances, focusing solely on features that passed a significance threshold in the Kruskal-Wallis test (*p*-value ≤0.05). ANCOM-BC analysis was conducted to detect differences between groups applying the Holm method for *p*-value adjustment. This analysis included only features present in at least 90% of samples, setting a convergence tolerance of 1e-05 and limiting the analysis to 100 iterations to minimize type I error rates. MaAsLin2 was utilized to identify associations with the group as a fixed effect, requiring a minimum feature prevalence of 90% and employing z-score standardization for data normalization. Because case and control cows were paired and shared the same environment, timing since treatment, parity, and diet, other covariates (random effects) were not included when using any of these analytical methods. Significantly different features (adjusted *p*-value ≤0.05) were noted if they were detected using at least two of the three methods. Pairwise comparisons were made between treatment groups at each time point as well as between stages of lactation. Random Forest (RF) with 5,000 decision trees was used to estimate the out-of-bag (OOB) error rate that allows for correctly classifying the sample groups based on the metabolite composition. RF was also used to predict features (clusters and components) based on the discriminatory levels between sample groups ranked by their mean decrease accuracy (MDA).

#### 2.6.2 Multi-omics analyses

Associations between the fecal microbiome and metabolome were examined by correlating the relative abundances of known metabolites (by library ID) with microbial taxa at the phylum and species levels and for antimicrobial resistance genes (ARGs), virulence genes, and microbial metabolic pathways across samples. Spearman correlations were calculated with the R package Hmisc (https://cran.r-project.org/web/packages/Hmisc/index.html); coefficients (ρ) >0.75 with *p*-values < 0.01 were filtered to construct networks with Gephi v.0.9.2 (Bastian et al., 2009).

**FIGURE 2:**
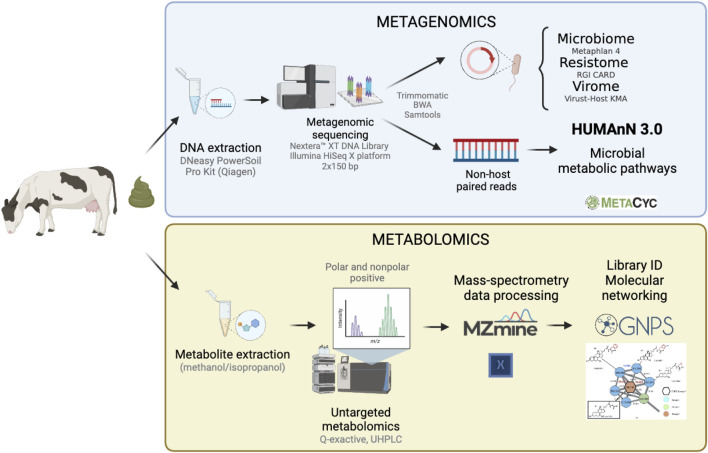
Summary of the methodology applied to analyze the functional gut microbiome of dairy cattle. Metagenomic sequencing (top panel) was used to characterize the microbial metabolic pathways, while metabolomics (bottom panel) was used to examine the metabolome composition among fecal samples collected from 40 dairy cows.

To characterize patterns of change in the abundance of microbial and metabolic features, hierarchical clustering was performed using the R package stats v4.1.2 (https://www.r-project.org/). Only those features that differed significantly between lactation stages using two of the three analytical methods (LefSe, ANCOM-BC and MaAsLin2) were included in this analysis. First, a distance matrix was constructed with the Euclidean metric using the fold-change (FC) relative to each feature average per sample. The FC was calculated by computing the mean abundance of each feature across samples, and by dividing the abundance of a feature from a given sample by their corresponding mean. The distance matrix was used for hierarchical clustering with the Ward method (ward.D) and the resulting tree was cut into 30 clusters. The optimal number of clusters was identified with the NbClust v3.0.1 package (Charrad et al., 2014), which resulted in five clusters; however, a finer analysis of each branch was biologically more meaningful. Boxplots of each hierarchical clustering group were constructed to visualize the patterns of change between stages of lactation. Experimental and analytical methods are summarized in Figure 2.

## 3 Results

### 3.1 Untargeted metabolomics reveals a diverse metabolite composition

Analysis of mass-spectra identified twice the amount of nonpolar (*n =* 11,007) metabolite clusters than polar (*n =* 5,390) clusters. Likewise, molecular networks aggregating metabolites based on their MS^2^ spectral similarity resulted in 1,122 nonpolar and 658 polar components (Supplementary Figure S1A). Network components connect clusters (nodes) that are structurally related via edges representing a modified cosine score that is calculated based on ions that differ by the mass difference (Supplementary Figure S1B). Only a small fraction of clusters had annotations based on library matches, corresponding to 2.48% of the total metabolites (polar, *n =* 135; nonpolar, *n =* 270), of which 68 were found with both polar and nonpolar modes.

**FIGURE 3:**
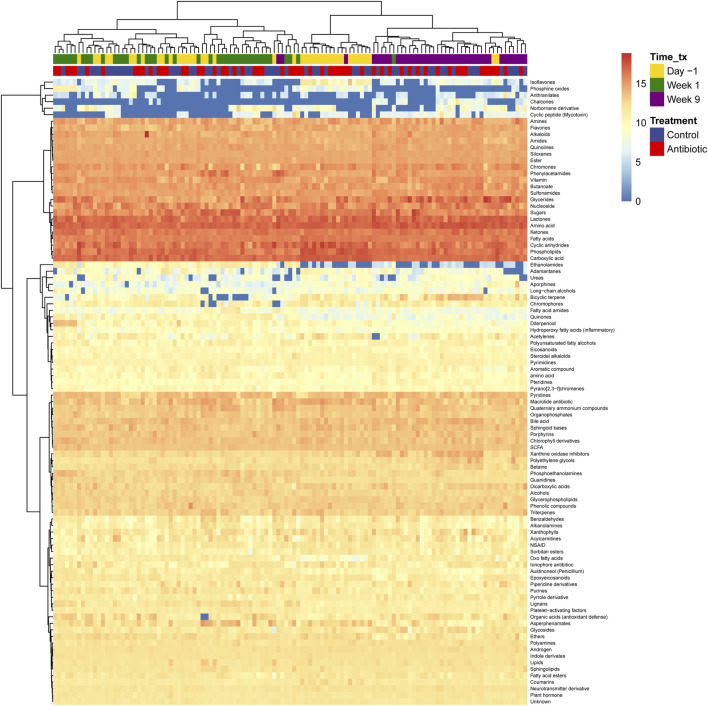
Fecal metabolome of dairy cows. The hierarchical clustering method Ward D2 was used to cluster rows (metabolites) and columns (samples) and only metabolites with library identification were included and aggregated at the class level. The color scale represents the logarithm (log) 10 of the relative abundance, with orange representing the most abundant metabolites and blue representing the least. Columns correspond to the samples, in which the time of collection (time_Tx) and IMM ceftiofur treatment status (Treatment) are indicated.

In the metabolite clusters with library matches, various classes of metabolites were observed in all samples (Figure 3). These included amino acids, lactones, carboxylic acids, cyclic anhydrides, phospholipids, glycerides, ketones, sugars, fatty acids, nucleosides, chromones, vitamins, and butanoate derivatives. Additionally, several highly abundant metabolites including guanosine, benzofuran-2-one, phthalic anhydride, phosphocholine, monoelaidin, anzacyclotridecan-2-one, cytidine, glycan lacto-N-biose, glycan lacto-N-biose, hexanedioic acid, propanoic acid, octadecenoic acid, and others, were found in all samples. A comprehensive list of the relative abundance of each metabolite and class detected is shown in Supplementary Table S2. Importantly, known metabolites categorized at the class level clustered together based on the stage of lactation but not the ceftiofur treatment status. The samples collected during the fresh period were most similar to each other.

### 3.2 Microbial metabolic pathways highlight the importance of essential molecule biosynthesis

Among the 159 samples, 262 metabolic pathways were identified that were assigned to bacterial taxa representing 797 pathways with different bacterial contributions. Only nine bacterial genera, however, were assigned to the pathways and included: *Bifidobacterium* (*n =* 75 pathways), *Clostridium* (*n =* 4), *Escherichia* (*n =* 25), *Methanobrevibacter* (*n =* 18), *Olsenella* (*n =* 2), *Ruminococcaceae* unclassified (*n =* 26), *Sarcina* (*n =* 12), *Turicibacter* (*n =* 12), other (*n =* 262), and unclassified (*n =* 200). On average, 93% of the reads were classified as unmapped and 6% as unintegrated for microbial metabolic pathways.

At the class level the most abundant microbial pathways were associated with the biosynthesis of amino acids, nucleoside and nucleotides, carbohydrates, and vitamins (Supplementary Figure S2). Aminoacyl-tRNA charging, fermentation and glycolysis pathways were also highly abundant in all samples. Pathways associated with the biosynthesis of fatty acid/lipids, cell structures, aromatic compounds, pentose phosphate and secondary metabolites were also common but were found in lower abundance. Similarly, pathways linked to the degradation of nucleoside/nucleotides as well as carbohydrates, carboxylates, and amine polyamines were commonly found (Supplementary Table S3). The abundance of microbial metabolic pathways varied between phases, with late lactation predominantly displaying elevated levels for most pathways. Nonetheless, no distinct clustering at the class level was evident among the lactation stages or by treatment status (Supplementary Figure S2).

### 3.3 Fluctuations in alpha diversity were observed across the sampling period

**FIGURE 4:**
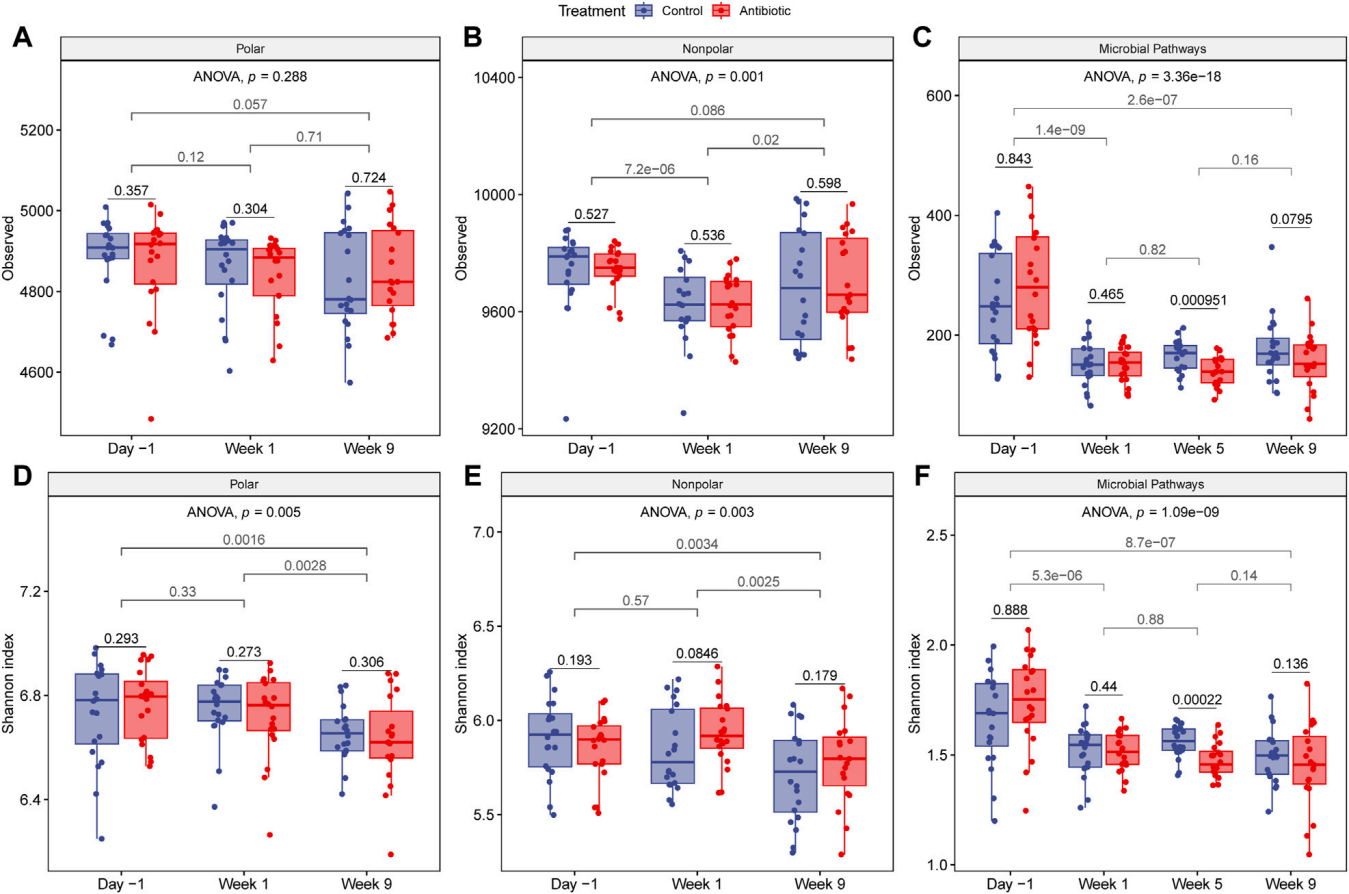
Alpha diversity of metabolites and microbial pathways. The top three panels show the number of observed features for **(A)** polar, **(B)** nonpolar, and **(C)** microbial metabolic pathways, while the bottom panels represent the Shannon index, for **(D)** polar, **(E)** nonpolar, and **(F)** microbial metabolic pathways, respectively. *p*-values were calculated with one-sided and paired *t*-test to compare treatment groups within a sampling point (black) or between time points regardless of treatment (gray). Each boxplot shows the median, lower, and upper quartiles with the whiskers representing extreme values in the distribution. Friedman’s test, which accounts for repeated measures, indicates significant fluctuations in alpha diversity over time.

The within-sample diversity was measured by comparing the number of observed features and the Shannon index for the metabolomes and metagenomes among samples collected from the same time point, between two time points, and over the entire sampling period while accounting for repeated measures. For the number of nonpolar metabolites, considerable fluctuations in alpha diversity were detected over time (ANOVA, *nonpolar*: *p* = 0.001). The number of polar metabolites, however, displayed a more consistent number of clusters (*Observed*, ANOVA, *p* = 0.288) but with varying evenness (*Shannon*, ANOVA, *p* = 0.005) (Figures 4A,D). A significantly greater number of nonpolar metabolites was detected during lactation (day -1) relative to the dry-off period (week 1) (*t*-test: *p* < 0.0001), though no difference was observed relative to the fresh period (*t*-test, *p* = 0.086). Fresh cows exhibited a similar number of metabolites as when they were dry (*t*-test, polar: *p* = 0.71, nonpolar: 0.98) but with lower evenness for polar (*Shannon*, *t*-test, *p* = 0.0028) and nonpolar (*Shannon*, *t*-test, nonpolar: *p* = 0.0025) metabolites (Figures 4B,E). Although the metabolite richness was similar between fresh and lactating cows, the Shannon index showed lower diversity in the fresh phase denoting a transition like the one detected for diet composition. No differences were observed between polar and nonpolar metabolites when comparing the ceftiofur-treated and control cattle at any of the time points.

For the metabolic pathway predications, fluctuations in alpha diversity were also observed across samplings (ANOVA, *p* < 0.0001) (Figures 4C,F). Interestingly, the alpha diversity was more similar between the dry and fresh cows than between fresh and lactating cows despite the similarity in diet. Compared to cows in the dry and fresh periods, cows in late lactation had a significantly greater quantity and diversity of microbial metabolic pathways (*t*-test, *p* < 0.0001). Stratifying by treatment status did not result in significant differences in alpha diversity between the groups, except that the number of metabolic pathways at week 5 was significantly higher in control cows as compared to the cows given IMM ceftiofur at dry off (*t*-test, *p* < 0.001). Because samples from week 5 were not analyzed with untargeted metabolomics, however, we could not compare between treatment groups at this time point. Nonetheless, no long-term effects in the number of metabolites were observed at week 9 (fresh cows).

### 3.4 Beta diversity of the metabolome and microbial pathways varies across samplings, whereas the effects of ceftiofur treatment manifest several weeks post-treatment

**FIGURE 5:**
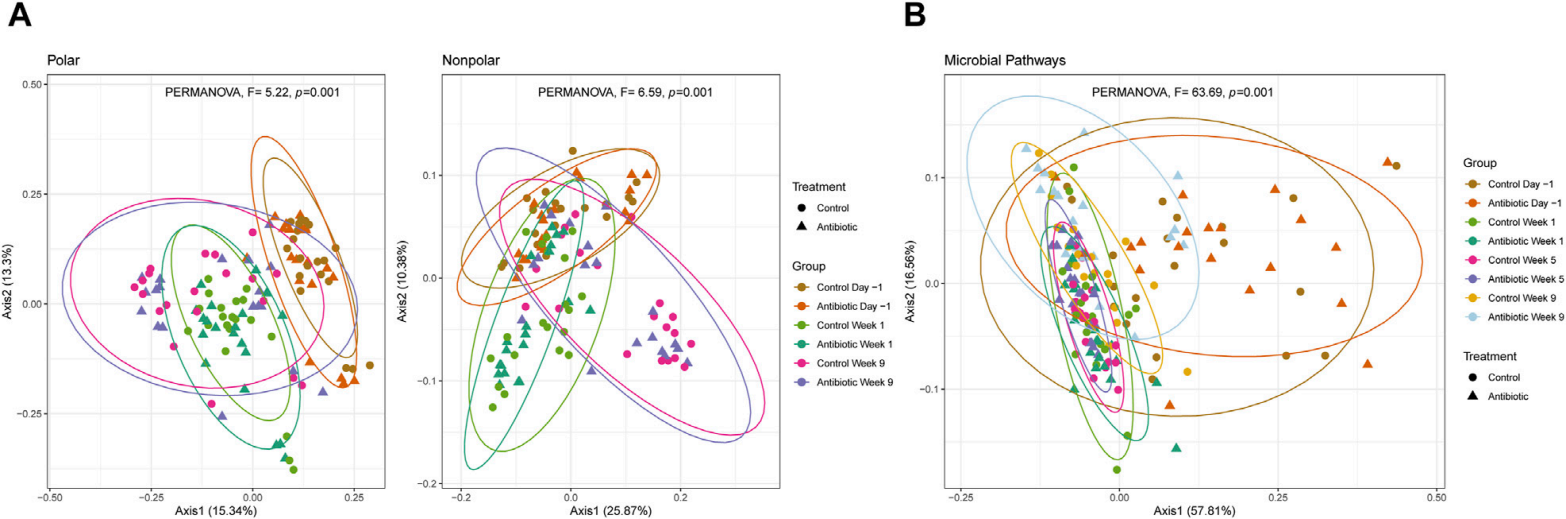
Beta diversity of **(A)** polar and nonpolar metabolites; and **(B)** microbial pathways. PCoA of the Bray-Curtis dissimilarity is clustered by treatment and sampling point (ellipses contain at least 90% of the samples in a group). Control animals are indicated by circles, whereas the ceftiofur-treated animals are indicated by triangles within each plot.

Bray-Curtis dissimilarity distances showed significant differences between samplings for polar and nonpolar metabolome composition comprising all metabolite clusters (PERMANOVA, *p* < 0.001) (Figure 5A). Although the microbial pathways had overlapping composition between dry and fresh cows (PERMANOVA, *p* > 0.3), the lactating cows showed a significantly higher dispersion in the PCoA compared to the samples from dry and fresh cows (PERMDISP, *F* = 53.32, *p* = 1.34e-11) as well as a different average composition (PERMANOVA, *F* = 63.69, *p* = 0.001) (Figure 5B). Despite the metabolome composition differences associated with the sampling period, the microbial metabolic pathways were similar in the dry and fresh phases. Furthermore, cows treated with IMM ceftiofur had an identical mean metabolite composition as the controls (PERMANOVA, *p* > 0.38) even though differences in the composition of the microbial pathways were observed in weeks 5 (PERMANOVA, *F* = 4.25, *p* = 0.007) and 9 (PERMANOVA, *F* = 2.67, *p* = 0.045).

### 3.5 Differences in the metabolome were observed between ceftiofur-treated and control cows at specific time points

After comparing the abundance of a total of 16,589 metabolite clusters, 3,753 metabolite components, and 797 microbial-metabolic pathways, only one metabolite cluster was significantly different between controls and cows treated with IMM ceftiofur 1 week after the treatment. This cluster corresponds to the nonpolar-positive metabolite cluster #6574 with a parent mass of 245.07 m/z, and a consensus retention time of 1.08 min (Supplementary Figure S3). This cluster is not identifiable and was not part of a network component, thereby limiting our understanding of its occurrence in the ceftiofur-treated animals. Similarly, RF could not correctly classify the metabolomic composition by treatment group at any time point; the OOB estimate of error rate was >55% when groups were compared based on metabolite or microbial-pathway composition at each time point.

**FIGURE 6:**
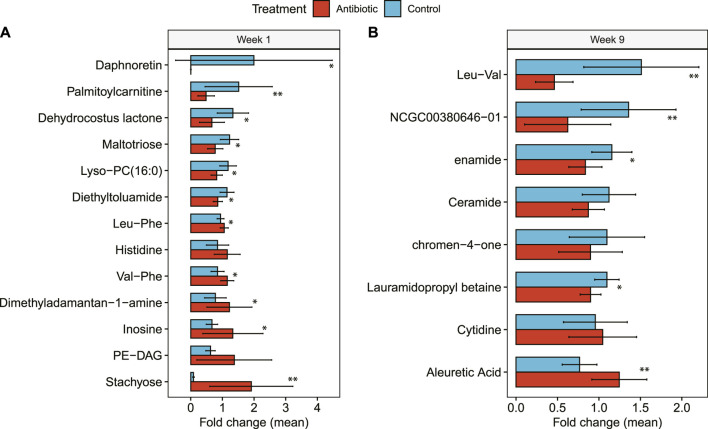
Hindgut metabolites identified in dairy cows with IMM ceftiofur (antibiotic) treatment relative to the control group. Differences in the relative abundance of each metabolite are shown at **(A)** 1 week after treatment (Week 1) and **(B)** 9 weeks after treatment (Week 9). The bars in the figure represent the mean fold change along with the corresponding confidence interval. One-tailed Wilcoxon signed-rank test was used to compare the relative abundance and significance (*p*-value) is shown as **≤0.01, or *≤0.05. NCGC00380646-01 represents (E)-5-(4-methoxy-5-methyl-6-oxopyran-2-yl)-3-methylhex-4-enoic acid.

Comparing groups with RF when considering only metabolite clusters with a library ID, which excluded unknown metabolites, the classifier error between treatment groups was reduced. Specifically, the OOB was 30% at day -1, 25% at week 1, and 18% at week 9. The primary identifiable metabolites that influenced the classification with RF varied across the time points. A week after drying off, for instance, inosine and palmitoylcarnitine were higher and lower, respectively, in cows treated with IMM ceftiofur (Mean decrease accuracy (MDA): 10.43, 7.83) than controls (Figure 6A). At week 9, the most important classifier was Leu-Val, followed by (E)-5-(4-methoxy-5-methyl-6-oxopyran-2-yl)-3-methylhex-4-enoic acid, which was higher in the controls relative to the antibiotic-treated cows (MDA: 14.31) (Figure 6B). By contrast, aleuretic acid (MDA: 7.49) was higher in the antibiotic-treated cows at week 9 compared to the controls.

### 3.6 Predicted microbial functions differed between ceftiofur-treated and control cows at specific time points

In the microbial metabolic pathway analysis, we identified nuanced distinctions among treatment groups. During the first week, for example, only seven microbial metabolic pathways exhibited disparities between cows treated with ceftiofur and the control group. These differences included an elevated presence of thiamine phosphate pathways by yeasts, as well as reduced levels of biosynthesis of cis-vaccenate and the degradation of punine ribonucleosides, D-fructuronate, and 4-deoxy-L-threo-hex-4enopyranuronate in the ceftiofur-treated cows.

**FIGURE 7:**
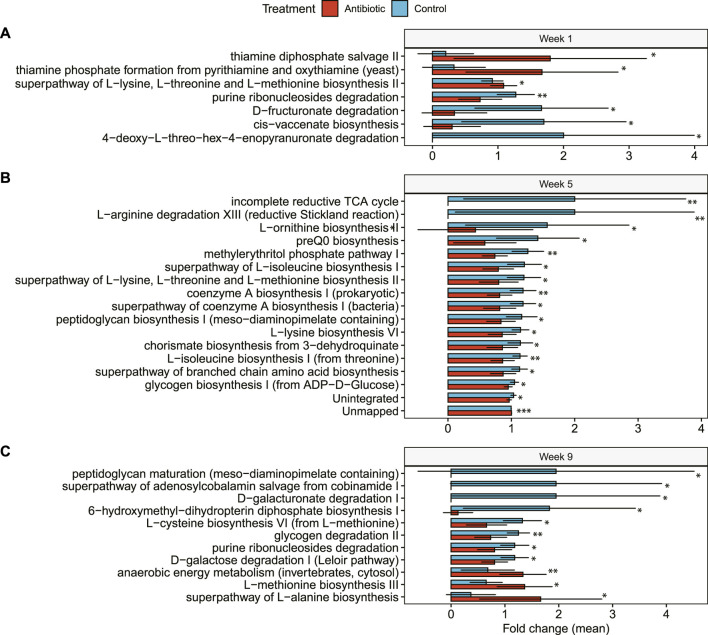
Differentially abundant microbial pathways in cows treated with IMM ceftiofur (antibiotic) compared to the control group. Differences in the relative abundance of each microbial pathway are shown at: **(A)** 1 week after IMM treatment (Week 1); **(B)** 5 weeks after treatment (Week 5); and **(C)** 9 weeks after treatment (Week 9). The bars in the figure represent the mean fold change along with the corresponding confidence interval. One-tailed Wilcoxon signed-rank test was used to compare the relative abundance and significance (*p*-value) is shown as **≤0.01, or *≤0.05.

At week 5, 17 microbial pathways were significantly less abundant in the cows treated with ceftiofur than the control cows; these included pathways involved in the biosynthesis of amino acids (i.e., L-ornithine, L-isoleucine, L-lysin, L-threonine, L-methionine), peptidoglycan, glycogen, isoprene, preQ0, chorismate, and coenzyme A, as well as in the degradation of L-arginine (Figure 7A). The unintegrated pathways of *Ruminococcaceae bacterium P7* and methylerythritol phosphate pathway I, however, were significantly lower in ceftiofur-treated cows. During week 5, several pathways were primarily identified in control cows but were absent in those given ceftiofur (Figure 7B). These pathways include L-arginine degradation XIII (controls, *n* = 5), the incomplete reductive TCA cycle (controls, *n* = 5), and L-ornithine biosynthesis II (controls, *n* = 6; ceftiofur, *n* = 1).

At week 9 (fresh phase), eleven microbial pathways explained differences between treatments using RF (Figure 7C). The most important differentially abundant pathways among the ceftiofur-treated cows included those related to peptidoglycan maturation, degradation of D-galacturonate, glycogen, purines, D-galactose, and the biosynthesis of 6-hydroxymethy-dihydropterin diphosphate and L-cysteine. Mirroring the observations from week 5, three microbial pathways were exclusive to controls (*n* = 4) at week 9, while another pathway was detected in just one ceftiofur-treated cow and seven controls. Curiously, a higher abundance of pathways related to anaerobic energy metabolism, and the biosynthesis of L-methionine and L-alanine were identified in cows treated with ceftiofur compared to the controls.

### 3.7 Differences in metabolites and microbial pathways were detected across samplings regardless of treatment status

The OOB error was 1.68% across the samplings, as the metabolomes of two fresh cows were misclassified as lactating cows. Hierarchical clustering of the 50 most important metabolite components identified with RF showed a transitional composition in fresh cows between the lactation stages (Supplementary Figure S4). Only four of these 50 components had clusters with library identification representing long-chain fatty acids, which were more abundant in the dry-off period (week 1), and amino acids that were increased during lactation (Supplementary Figure S5).

Differential abundance tests also identified 9,850 features that differed between samplings, corresponding to 46.59% of the total features (metabolites and microbial pathways) (Supplementary Figure S6). Dry and fresh cows showed a lower number of different features (53.88% of the total) than lactating and dry and cows (64.64%) or lactating and fresh cows (66.9%). In particular, the dry and fresh cows only differed in a few microbial pathways, whereas approximately one-third of the microbial metabolic pathways had varying abundances during lactation as compared to the other stages.

**FIGURE 8:**
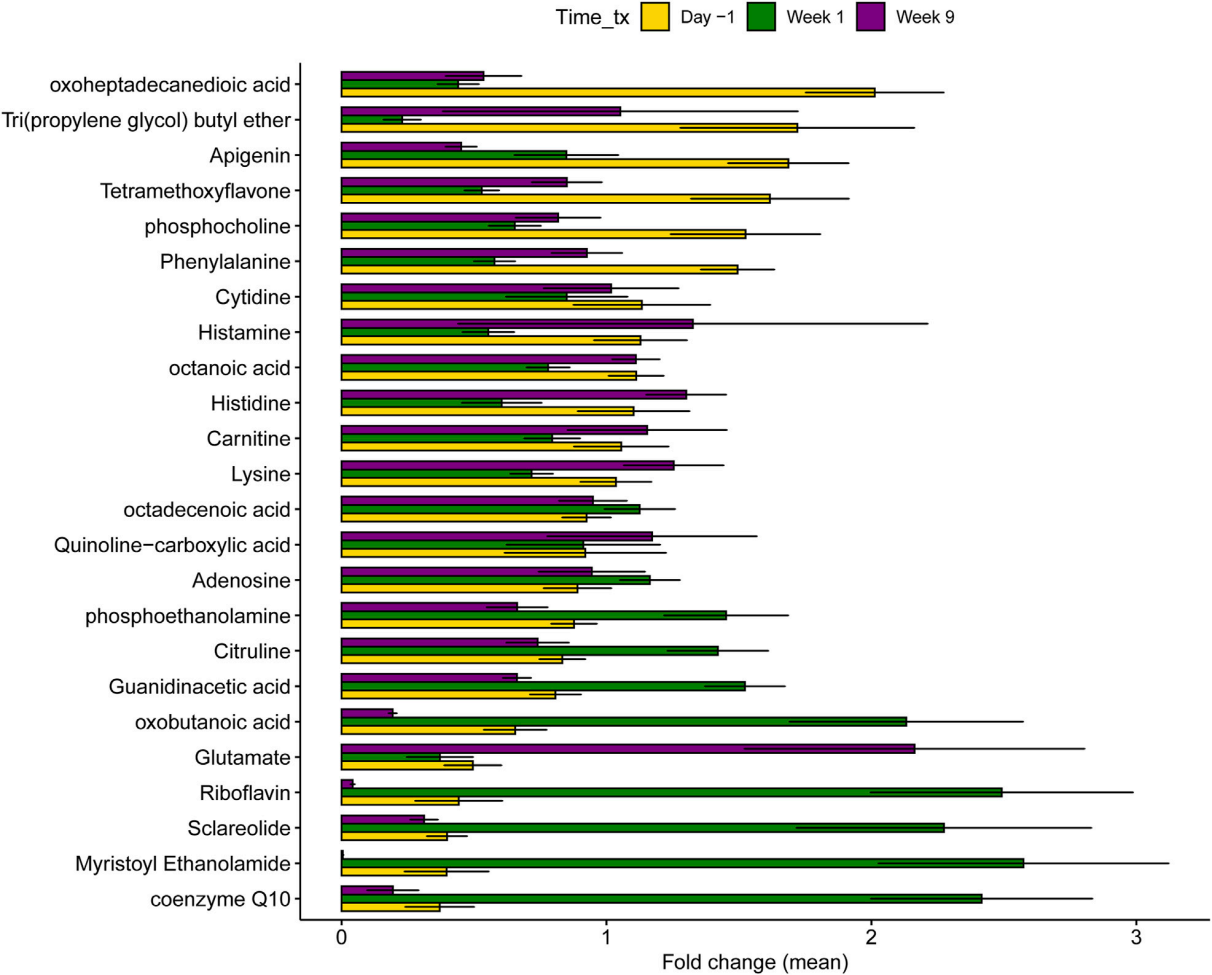
Differentially abundant metabolites across the three samplings in all 40 dairy cows regardless of treatment status. Each sampling corresponds to different stages of lactation. Day -1 corresponds to late lactation, Week 1 to dry off, and Week 9 to fresh cows. Data from all animals was combined and not stratified by treatment status. The bars in the figure represent the mean fold change along with the corresponding confidence interval.

**FIGURE 9:**
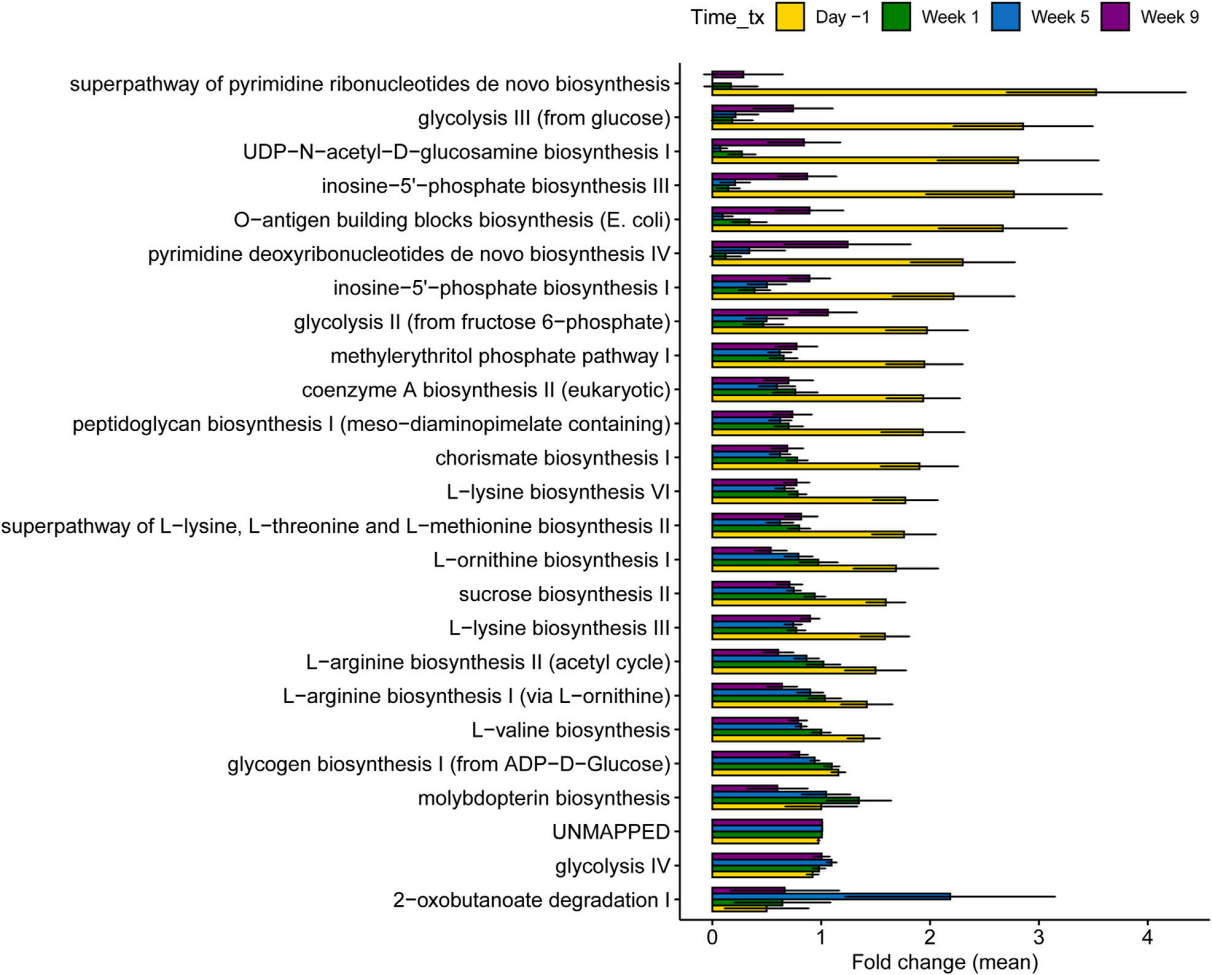
Differentially abundant microbial pathways across samplings in 40 dairy cows regardless of treatment status. The different samplings are shown and correspond to the different stages of lactation. Day -1 represents late lactation, while Week 1 and Week 9 represent the dry-off and fresh periods, respectively. The bars in the figure represent the mean fold change along with the corresponding confidence interval.

Among the top 25 most important metabolites classified by RF with library matches (Figure 8), cows in late lactation had a greater abundance of oxoheptadecanedioic acid, tri(propylene glycol) butyl ether, apigenin tetramethoxyflavone, phosphocholine, and phenylalanine compared to dry and fresh cows. Conversely, dry cows had higher concentrations of coenzyme Q10, myristoyl ethanolamide, riboflavin, harmol, oxobutanoic acid, guanidineacetic acid, citrulline, and phosphoethanolamide. Fresh cows demonstrated increased abundance of glutamate, histidine, and lysine, alongside histamine, which was elevated in both fresh and lactating cows compared to dry cows. Similarly, an evaluation of the top 25 microbial metabolic pathways showed that multiple pathways were significantly higher in lactating cows compared to those cows in the other stages (Figure 9). Pathways involved in the biosynthesis of nucleotides, amino acids, cell wall and glycolysis are among the most abundant. Intriguingly, during dry-off (week 5), higher levels of 2-oxobutanoate degradation I pathways were detected.

Notably, the top 8 most important pathways that predicted the sampling period through RF corresponded to three categories: 1) cell division, 2) amino acid biosynthesis, and 3) carbohydrate biosynthesis. These pathways were significantly higher during lactation, particularly those related to cell division that were mostly absent during the dry-off period. Cell-division pathways included inosine-5′-phosphate biosynthesis III, pyrimidine deoxyribonucleotides *de novo* biosynthesis IV, UDP-N-acetyl-D-glucosamine biosynthesis I, and O-antigen building blocks biosynthesis (*E. coli*), which were assigned to *Bifidobacterium*, *Turicibacter*, *Olsenella*, and *Escherichia coli* (Supplementary Figure S7). The three main pathways related to amino acid biosynthesis involved the superpathway of L-lysine, L-lysine biosynthesis VI, L-valine biosynthesis, and L-threonine and L-methionine biosynthesis II (Supplementary Figure S8). During lactation, these amino acid biosynthesis pathways were mainly assigned to *Bifidobacterium* spp. No taxa could be assigned to these pathways in the samples collected during the dry and fresh periods. Finally, carbohydrate biosynthesis pathways included glycogen biosynthesis I (from ADP-D-Glucose), sucrose biosynthesis II, and horismite biosynthesis I (Supplementary Figure S9). Although *Sarcina* was the main taxa assigned to glycogen and sucrose biosynthesis during the dry-off and fresh periods, *Bifidobacterium* was mainly associated with carbohydrate biosynthesis during lactation.

### 3.8 Multi-omics analysis identifies correlations between the microbiome and metabolome

Positive Spearman’s correlations among metabolites, microbial pathways, microbial species, viruses, and antimicrobial resistance genes were analyzed. Potential functional relationships between metabolites and microbial species included uncultured Firmicutes and Bacteroidetes species with triacylglycerol (TAG); *Clostridium* with N,N,N-trimethyllysine, N,N-dimethyldodecylamine N-oxide and myristamidopropyl betaine; Proteobacteria and *Blautia* with histamine; monolinolenin, beauvericin [+NH4+], and neobavaisoflavone, with *Campylobacter*, Ruminococcaceae uncultured bacteria (GGB3236), and bacteriophages from enterobacteria (Vectrevirus Vec3); 2′-deoxyadenosine with Gammaproteobacterial; 1,2-Diheptadecanoyl-sn-glycero-3-phosphocholine with uncultured Bacteroidetes. Fruchterman Reingold networks show other correlations between the microbiome and polar metabolites (Supplementary Figure S10) as well as nonpolar metabolites (Supplementary Figure S11).

### 3.9 Metabolome and microbiome patterns changed across the sampling period

**FIGURE 10:**
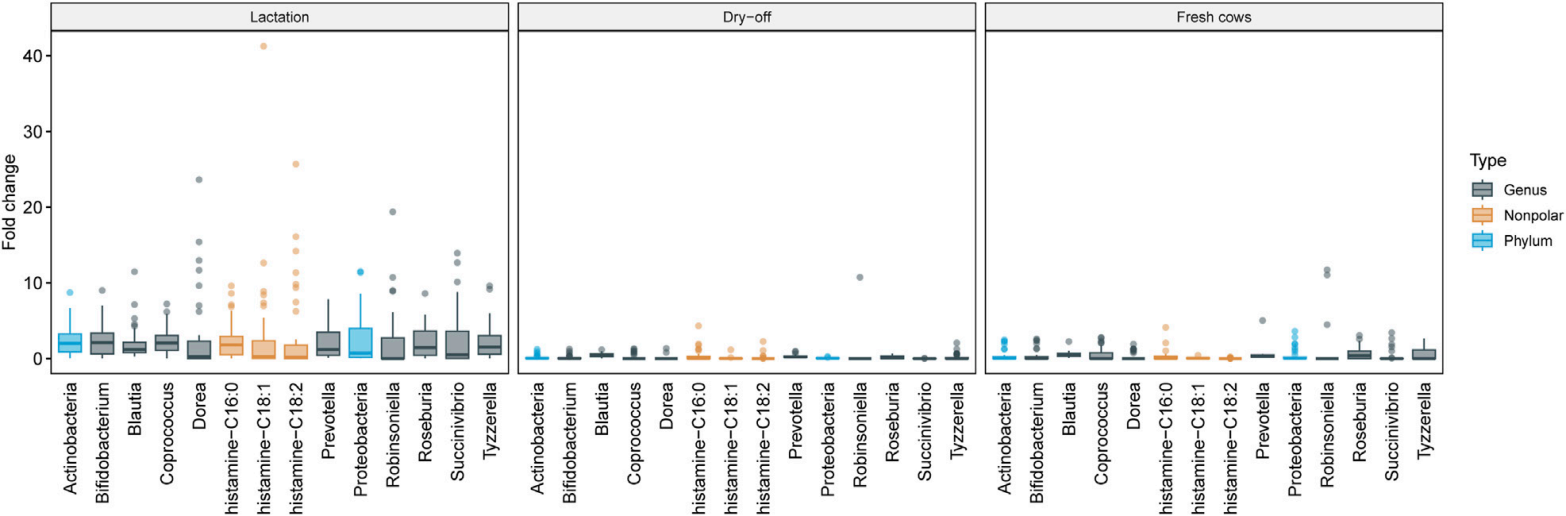
Metagenome and metabolome patterns by sampling period. This figure shows the variation in metagenome feature abundance at the genus and phylum levels as well as metabolome feature abundance across the sampling period. The samplings correspond to the Lactation (left panel), Dry-off (middle panel), and Fresh (right panel) periods. Emphasis is placed on features that are dominant in one sampling but nearly absent in others. These patterns were discerned using Hierarchical Clustering with the Ward D2 method. The *Y*-axis details the fold-change, compared to the average feature abundance across all samples. Boxplots encapsulate the median and interquartiles with whiskers indicating extreme values and marked outliers also presented.

To better explore the functional associations of differentially abundant features, hierarchical clustering was performed with only those known metabolic clusters that differed significantly between samplings using LefSe, MaAsLin2 and ANCOM-BC. A tree was constructed based on a distance matrix highlighting the fold-change of metabolites and metagenomic features that differed significantly (*n* = 684). One relevant group showed concomitant higher levels of Actinobacteria, Proteobacteria, and histamine during lactation (Figure 10). This finding suggests a role of these taxa in the production of pro-inflammatory compounds such as histamine, which was also observed in the correlation networks.

Fresh cows showed higher levels of urate and nonpolar plant-derived compounds, which was likely due to a diet rich in alfalfa hay that was provided to the animals during this phase. Other clusters also showed patterns with lower contrast across the stages of lactation, with the most relevant HC groups displayed in Supplementary Figure S12. For instance, higher quantities of amino acids and dipeptides were detected with the polar mode on samples from lactation, which was related to a higher abundance of bacterial amino acid synthesis pathways mentioned priorly (Supplementary Figure S8). Not surprisingly, higher levels of androstane were also detected in fresh cows, which are expected to have peak levels of estrogens at this time (Supplementary Figure S13).

## 4 Discussion

In this study, we sought to identify changes in the gut metabolome of dairy cows due to IMM treatment with ceftiofur applied at dry-off over a 9-week period. Although IMM ceftiofur treatment impacted the abundance of specific metabolites, substantial alterations in the overall metabolome composition were not observed and could be attributed to low levels of ceftiofur metabolites in the cattle gut. Indeed, ceftiofur metabolites were not detected in the feces of IMM-treated cows 1-week after treatment. This was an anticipated finding as a prior study of steers treated subcutaneously found the total concentration of ceftiofur equivalents to be negligible in the gastrointestinal tract after 96 h (Foster et al., 2019). Another study showed that ceftiofur excretion began as early as 24 h after intramuscular administration, but most residues were detected in the urine (60%–80%) (Brown et al., 1991). Therefore, the lack of detection of ceftiofur metabolites in our samples post-treatment likely contributed to fewer functional alterations in the gut. Regardless, several metabolites were detected in greater abundance in the ceftiofur-treated *versus* -untreated cows along with an uncharacterized metabolite, which was found exclusively in the treated cows 1-week after treatment. This metabolite could represent a constituent of Spectramast^®^ DC, which also contains microcrystalline wax, oleoyl oilyoxyglyceride, and cottonseed oil as well. Additional studies are needed for verification.

Among the metabolites present in greater abundance in the ceftiofur-treated cows was stachyose, an oligosaccharide of plant origin that is resistant to host enzymatic digestion (Zheng et al., 2000). Its presence suggests potential disruptions in gut bacteria responsible for its fermentation. Additionally, the enhanced levels of PE-DAG and inosine detected in the ceftiofur-treated cows suggest variation in microbial metabolism of lipids, energy, and cellular signaling, while lower concentrations of anti-inflammatory compounds, daphnoretin and dehydrocostus lactone (He et al., 2002; Chen et al., 2020), were discerned. Diminished fatty acid oxidation processes, as reflected by the reduced levels of palmitoylcarnitine, phospholipids, and trisaccharide maltrotiose in the ceftiofur-treated cows, also suggest differences in digestive processes compared to the controls.

Microbial metabolic pathways were also modified following IMM ceftiofur treatment. While the impact was not immediately observable in these pathways 1-week after treatment, discernible functional alterations were detected by week 5. Indeed, the diminished abundance of numerous microbial metabolic pathways at week 5 suggests a curtailed metabolic potential of the bovine microbiome in response to ceftiofur treatment. Notable among these were decreased utilization and biosynthesis of certain amino acids encompassing L-arginine, L-ornithine, L-isoleucine, L-lysine, L-threonine, and L-methionine. Furthermore, the capacity to produce precursor molecules and the synthesis of crucial biochemicals such as sterols, carotenoids, chlorophylls, fatty acids, cholesterol, among others, was decreased in the ceftiofur-treated group. Since the metagenomic analysis did not invariably elucidate the activity of the gene-encoded enzymes present in fecal samples in the first week post-treatment, the application of metatranscriptomics could be used in future studies to provide clarification.

Importantly, ceftiofur treatment had no short-term or persistent effects on metabolome diversity in the cow gut over the 9-week sampling period. Although this result is consistent with our findings showing no difference in microbiome diversity in the same cohort of cows (Vasco et al., 2023), the ceftiofur-treated microbiome showed a reduced propensity for energy production. Evidence for this observation is provided by the observed downturn in the biosynthesis of glycogen and coenzyme A. By the ninth week, however, multiple pathways associated with *Bifidobacterium* were more abundant in ceftiofur-treated cows. For example, L-methionine and L-alanine, which are prominently involved in anaerobic energy metabolism and the biosynthesis of amino acids, are associated with the genus *Bifidobacterium*, a member of the phylum Actinobacteria that was significantly more abundant in ceftiofur-treated cows previously (Vasco et al., 2023). Despite these observations, a decline in sugar degradation (such as D-galactose, purine ribonucleosides, glycogen, D-galacturonate) and a reduction in the production of vitamin B12 and peptidoglycan maturation were observed in the ninth week following ceftiofur treatment. These findings highlight the complex and multilayered response of the bovine gut microbiome to IMM ceftiofur treatment.

While we did not collect milk samples for evaluation in this study, it is worth noting that treatment-associated modifications in the gut microbiome and metabolome could potentially impact milk quality. The presence of *Bifidobacterium*, for example, has been linked to higher milk-fat yields (Jami et al., 2014), while genus *Prevotella* (phylum Bacteroidetes) has been connected to metabolic pathways integral to protein and fat content in milk as well as the production of volatile fatty acids (Wu et al., 2021). Despite these associations, the primary determinants of milk composition are specific to each herd’s diet (Albonico et al., 2020) and to a lesser extent, the interplay between genetic makeup and the composition of rumen bacteria (Buitenhuis et al., 2019). It is important to recognize that even though IMM ceftiofur may exert specific effects on the fecal metabolome and the functionality of the microbiome, the judicious application of antibiotic therapy during the dry-off period is essential for preventing mastitis. This condition not only impairs milk production but also has adverse consequences on the welfare of the animal (Ruegg, 2017). We did not assess the efficacy of IMM ceftiofur in preventing IMM infections herein, nor did we investigate whether IMM infections induce inflammation and independently perturb the gut microbiota.

Moreover, our analysis demonstrated that the overall metabolome and related microbial metabolic pathways varied across the sampling period. Each stage of lactation, which is characterized by unique physiology and diets, had a distinct metabolome and functional microbiome, highlighting the collective role that these factors play in metabolome variation regardless of ceftiofur treatment. Even though the individual impact of these factors could not be explored based on our study design, prior studies have shown that diet impacts the fecal metabolome and microbiome composition in cows (Zhang et al., 2018; Hagey et al., 2019; Liu et al., 2020; Vasco et al., 2021). For instance, increasing grain-forage ratios have been linked to a higher abundance of Proteobacteria and a lower abundance of Bacteroidetes in feces (Zhang et al., 2018; Liu et al., 2020). Moreover, diets with >30% grain given to cows in early lactation significantly changed the ruminal metabolome, increasing the abundance of short-chain fatty acids as well as toxins, inflammatory compounds, putrescine, methylamines, and ethanolamine (Saleem et al., 2012). Herein, cows in late lactation (day -1) received the highest amount of grain in the diet, constituting about 39% of the dry matter intake vs 26% in fresh cows (week 9) and 7% in dry cows (week 1). It is therefore likely that different feed ingredients will have distinct effects on the metabolome and microbial activity in the bovine gastrointestinal tract.

Comparatively, those cows sampled during late lactation also had enhanced diversity of microbial pathways and metabolites. Phenylalanine, for instance, is an essential amino acid, and had higher levels during late lactation. This finding suggests increased protein intake or metabolism in cows on the maintenance diet compared to the diets used during other stages of lactation (Reitelseder et al., 2020). It also aligns with the diet formulation containing higher levels of crude protein compared to those administered in the early dry and fresh phases. Similarly, compounds like phosphocholine and carnitine are involved in lipid metabolism and energy production; hence, the higher levels observed during late lactation may reflect differences in energy substrate utilization compared to dry and fresh phases. Higher levels of dry matter, fat, net energy, non-fiber carbohydrates, starch and vitamin A were also provided during lactation that could have differentially impacted community function and require further examination.

During late lactation when a higher abundance of Actinobacteria and Proteobacteria was observed, greater levels of histamine were also found. Microbial-origin gut histamine, which is linked to grain-rich diets, has been associated with inflammatory responses, such as laminitis (Garner et al., 2002), and inflammatory reactions in the bovine lung (Barcik et al., 2019). Furthermore, increased levels of histamine in the gut can lead to symptoms such as increased vascular permeability, edema, and the recruitment of inflammatory cells (Ashina et al., 2015). These outcomes can result in the translocation of bacteria, toxins, and other molecules across the intestinal barrier, potentially contributing to inflammatory processes and other gut issues.

During the dry-off period, cows are typically transitioned to a diet primarily composed of forage with a lower percentage of grain (Dancy et al., 2019), which could impact the metabolic response. For example, propionic acid is a volatile fatty acid produced during rumen fermentation of carbohydrates, and its higher levels may reflect increased fermentation of forage-based diets, particularly grasslage (Ribeiro et al., 2009). Compounds like citrulline, guanidinacetic acid, oxobutanoic acid, riboflavin, myristoyl ethanolamide, and coenzyme Q10 are involved in energy metabolism, amino acid metabolism, and cellular processes, and their levels may be influenced by metabolic shifts. Similarly, during dry off, cows also exhibited a higher abundance of the 2-oxobutanoate degradation I pathway, which plays a role in the breakdown of specific amino acids and contributes to energy production and the generation of key metabolic intermediates. Since dry-off is associated with changes in hormone levels, particularly the decline in lactation-related hormones such as prolactin (Ollier et al., 2013), hormonal fluctuations can also impact metabolic pathways and the production or utilization of certain compounds.

A higher diversity of microbial pathways, as was observed during late lactation, has also been associated with enteric infection in monogastrics in a case-control clinical study performed by our group (Hansen et al., 2024). Despite the similarity in diet and the metabolome between the fresh and lactating cows, the microbial metabolic pathway diversity and composition were significantly different. Hence, it is essential to emphasize the marked disparity in dry matter intake and metabolic status between fresh and lactating cows, particularly when distinguishing between negative and positive energy balances. During early lactation, cows typically experience a negative energy balance, whereas those in late lactation transition to a positive energy balance (Butler and Smith, 1989). In fact, the pathway profiles of fresh cows were like those observed in the dry phase suggesting a slow adaptation to a high grain diet. Although it only took a week on a forage-based diet at dry-off to identify changes in the functional gut microbiome, this was accompanied by lower levels of histamine-producing bacteria compared to the lactation stage. Since the core microbiome composition is unique to each farm due to factors that include housing, breed, and age (Hagey et al., 2019; Furman et al., 2020), changes in the diet are the most impactful on the cattle metabolome and microbial diversity. Although manipulation of the functional microbiome through dietary changes is plausible, functional changes can take longer to develop in a new environment as was observed herein. Nonetheless, it is important to note that we did not control for diet or other factors when making comparisons across sampling periods and hence, these relationships require validation in future studies.

Similar to findings from other metabolomic studies (da Silva et al., 2015; Peisl et al., 2018), many of the metabolites and microbial-metabolic pathways were unknown and could not be classified. Despite this limitation, biologically important compounds and metabolic pathways enabled the interpretation of some associations that were observed between the microbiome and metabolome. Future studies, however, should include GC/MS to promote the identification of short-chain fatty acids (SCFAs) since they have been linked to health outcomes in humans (Tan et al., 2014) and production in cattle (Bionaz et al., 2020). Moreover, associations between metagenome, metabolome and milk production could guide improvements in diet formulations, health, and probiotic development. Serum metabolome analyses could also help identify relationships between microbiome functionality and host factors such as hormonal levels (i.e., estrogens, cortisol, progesterone, prolactin), or metabolic disorders in cattle. Neither SCFAs or serum metabolites were evaluated in our study nor was the application of fecal proteomics, which could be used to define markers of immunity and inflammation that are indicative of specific host responses. These analyses as well as the use of metatranscriptomics and metaproteomics combined with targeted metabolomics should be performed in the future to better characterize the functional microbiome (Van Den Bossche et al., 2021).

## 5 Conclusion

IMM ceftiofur treatment of dairy cattle at dry-off resulted in alterations to the microbial metabolic pathways and fecal metabolites associated with lower biosynthesis of amino acids and energy a week after its application. Nevertheless, these alterations were not as pronounced as those observed with dietary changes and physiological shifts linked to lactation stage. Indeed, each stage of lactation was characterized by a distinct metabolome composition that was related to feed ration and physiology regardless of treatment status. During lactation, a higher level of microbial activity, particularly amino acid biosynthesis, was observed as compared to dry and fresh cows; however, histamine-producing bacteria were more abundant during late lactation. Together, these data highlight how integrative analyses of metagenomics and untargeted metabolomics data can be used to define the metabolite-microbe interactions in the cattle gut. Understanding the role of the gut environment in the microbial profile is critical to identify factors related to cow health in dairy farms.

## Data Availability

The paired-end metagenome raw reads used in this study are deposited in the NCBI repository, BioProject PRJNA825520 (Biosamples SAMN27520269 to SAMN27520427). FBMN data processed through GNPS is available online for polar and nonpolar metabolites (https://gnps.ucsd.edu/ProteoSAFe/status.jsp?task=d4a761f0a6be422c8b89db9408f57b0d and https://gnps.ucsd.edu/ProteoSAFe/status.jsp?task=1d6f7e95d2f04f96a94fede8c195702d, respectively). Additional analyses that support our conclusions are available in the GitHub repository (https://github.com/karla-vasco/metabolome_microbiome_cattle).
