# Supplementary material for: Untargeted metabolomics and metagenomics reveal signatures for intramammary ceftiofur treatment and lactation stage in the cattle hindgut
Source: Front Mol Biosci. 2024 May 21;11:1364637. doi: 10.3389/fmolb.2024.1364637 (PMC11148447; doi:10.3389/fmolb.2024.1364637)
Supplement: Supplementary file 1 [file DataSheet1.pdf]

Supplementary Figures

Untargeted metabolomics and metagenomics reveal signatures for intramammary ceftiofur treatment and lactation stage in the cattle hindgut

Karla A. Vasco[1*†], Zoe A. Hansen[1†], Anthony L. Schilmiller[2], Bailey Bowcutt[1], Samantha L. Carbonell[1], Pamela L. Ruegg[3], Robert A. Quinn[4], Lixin Zhang[1,5] and Shannon D. Manning[1*]

[1]Department of Microbiology, Genetics and Immunology, Michigan State University, E. Lansing, MI 48109, USA

[2]Research Technology Support Facility, Mass Spectrometry and Metabolomics Core, Michigan State University, E. Lansing, MI 48109, USA

[3]Department of Large Animal and Clinical Sciences, Michigan State University, E. Lansing, MI 48109, USA

[4]Department of Biochemistry and Molecular Biology, Michigan State University, E. Lansing, MI 48109, USA

[5]Department of Epidemiology and Biostatistics, Michigan State University, E. Lansing, MI 48109, USA

*** Correspondence:**
Corresponding Authors
karlitavasco87@gmail.com; mannin71@msu.edu

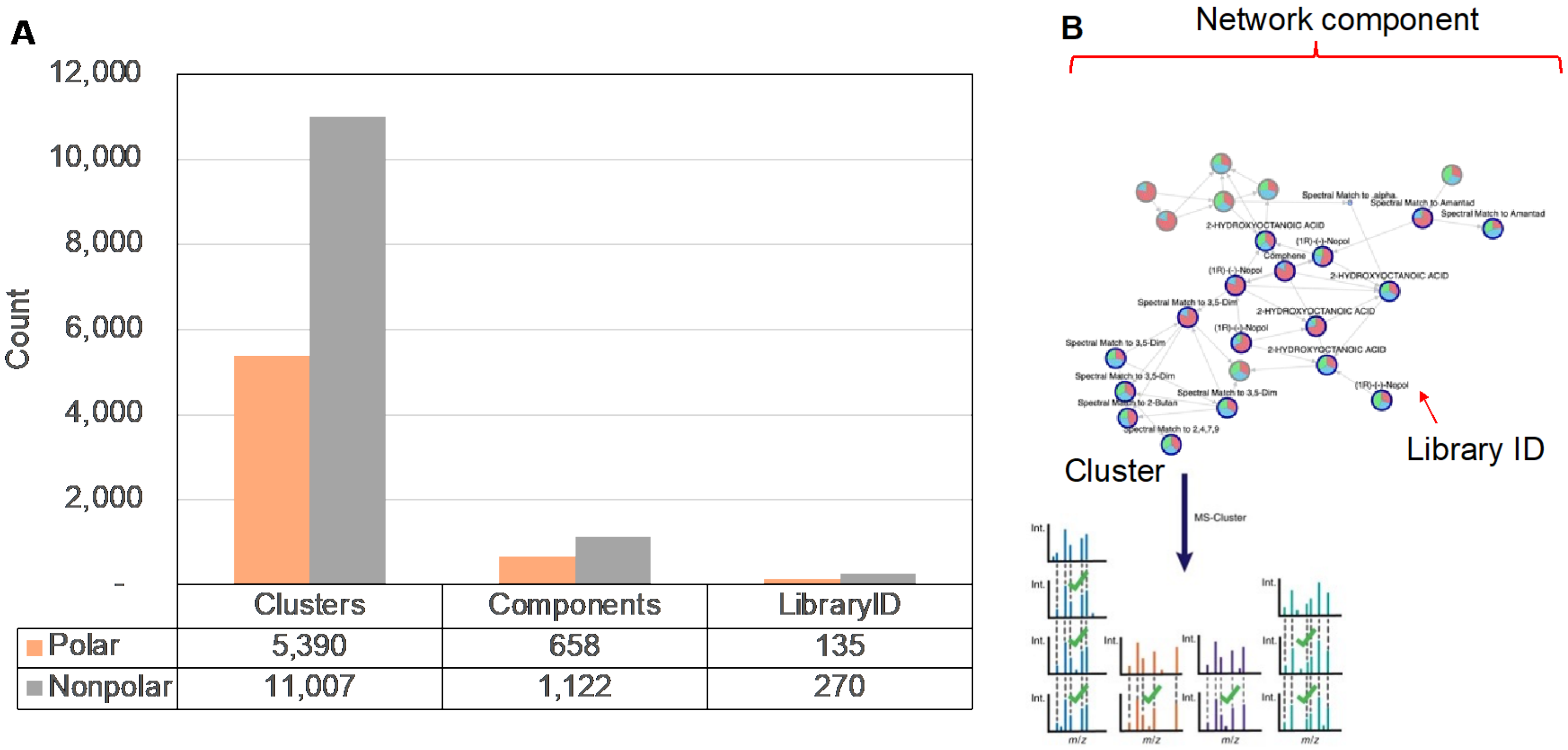

Supplementary Figure S1. Quantitative analysis of metabolite clusters, network components, and metabolites. A) Bar graph detailing the quantity of clusters discerned using MZmine, components collated using FBMN, and clusters assigned with library IDs through GNPS pipelines. B) Schematic representation of a network component comprising both identified and unidentified clusters. Each cluster corresponds to consensus MS^2 spectra from identical compounds detected across samples. Accompanying this, a graphical depiction of MS/MS spectra illustrates the aggregation process of these clusters.

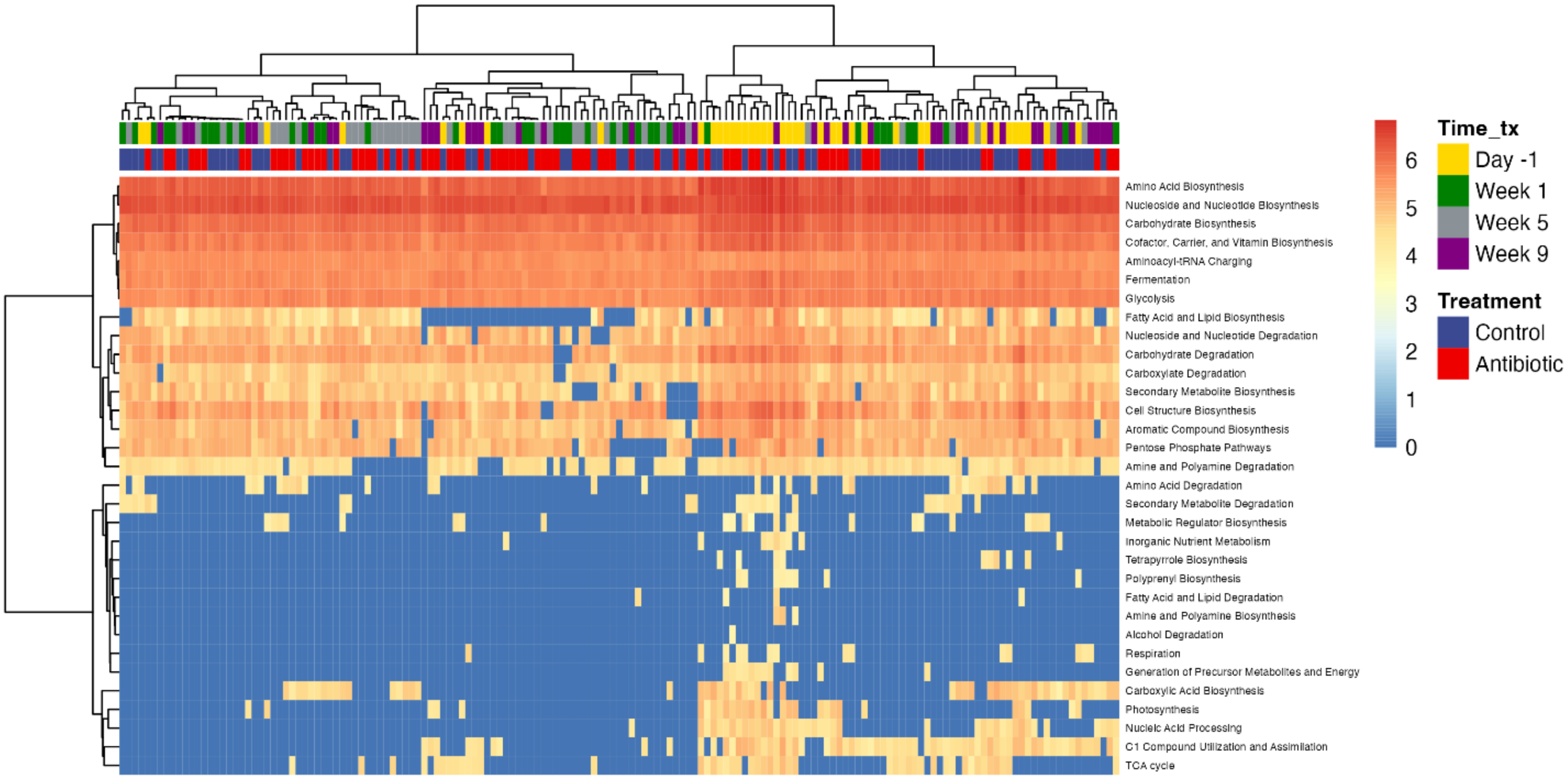

Figure S2. Microbial metabolic pathways identified in the fecal metagenome of dairy cattle. The hierarchical clustering method Ward D2 was used to cluster rows (microbial pathways) and columns (samples). Metabolic pathways were aggregated at the class level using MetaCyc classifications. The scale represents the logarithm 10 of the relative abundance. Columns represent samples, which are identified based on the time of collection (Time_tx) and IMM ceftiofur treatment status (Treatment).

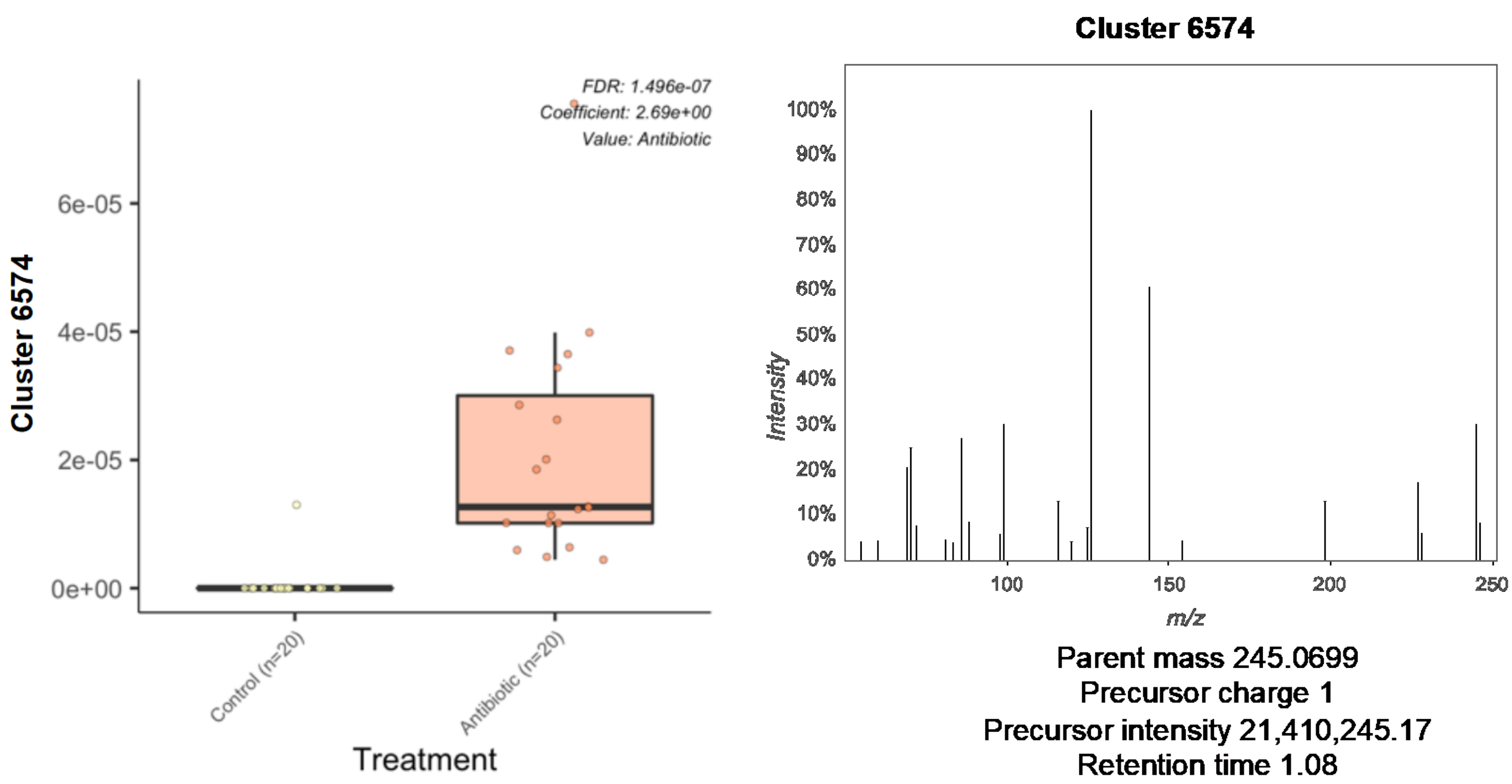

Figure S3. Cluster 6574 significantly different between treatments in week 1. A) Box plot based on the relative abundance per sample. B) Mass spectra of the unknown cluster.

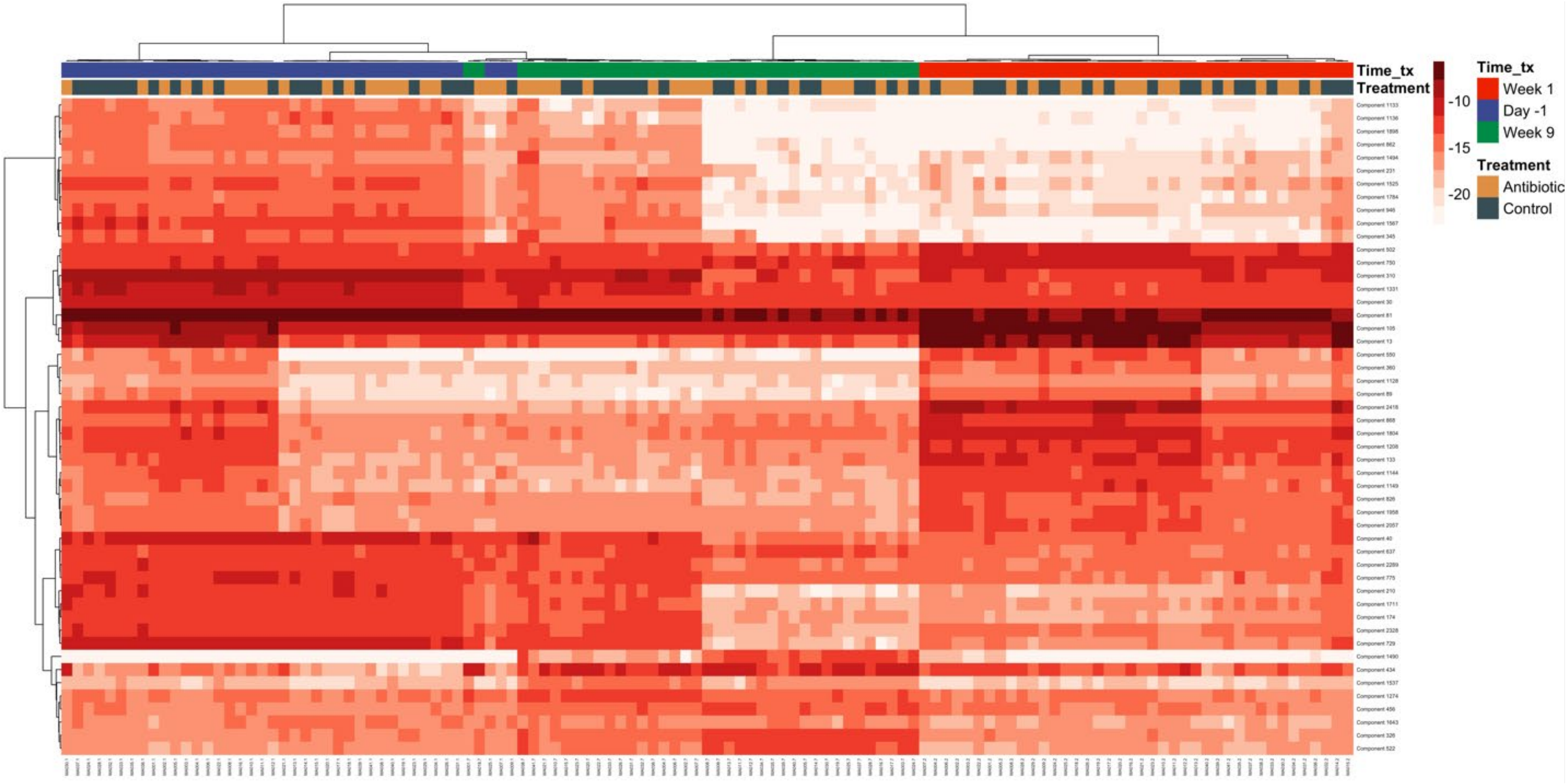

Figure S4. Heat map of the 50 most important nonpolar metabolite components for classifying samples by time point. Hierarchical clustering with the method Ward D2 was used to aggregate samples and components.

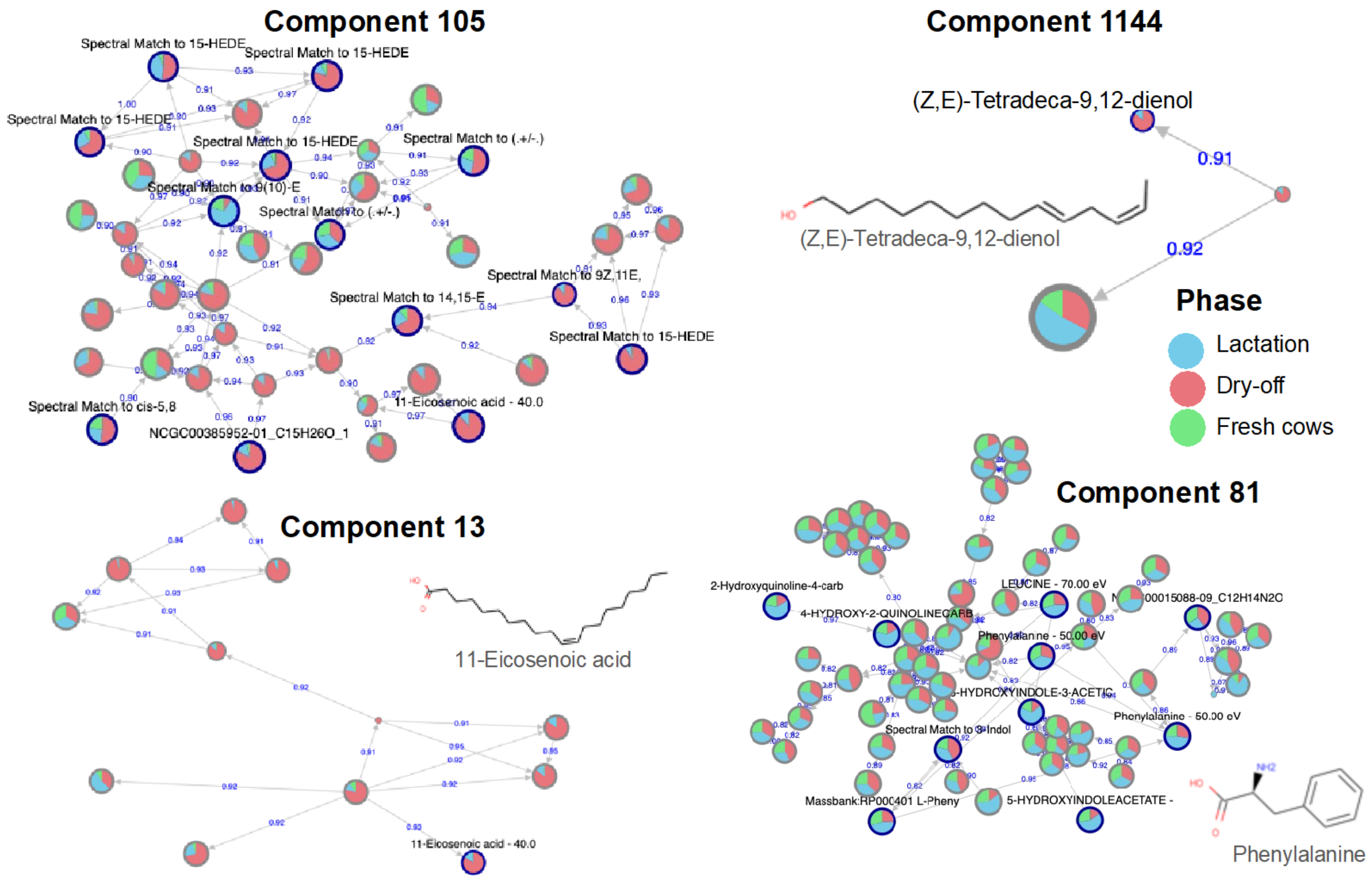

Figure S5. Components containing clusters by library ID that were among the 50 most important features for classifying the metabolome by lactation phase with RF. Edge numbers represent the cosine score of spectral similarity between two fragmentation spectra. The size of the clusters represents the number of files.

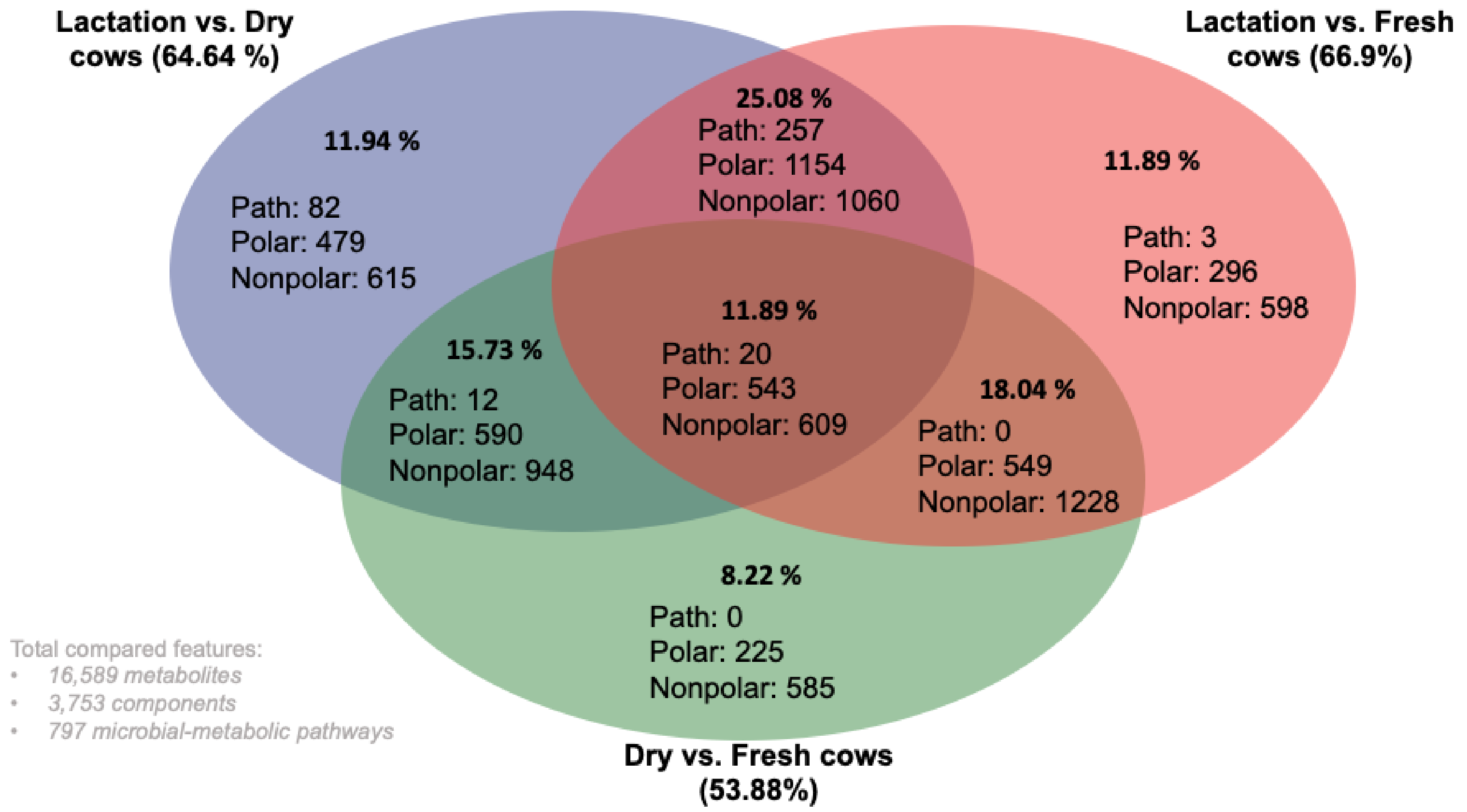

Figure S6. Venn diagram showing the number of biomarkers shared between lactation phases. Percentages represent the proportion of the total differential features. The percentage of features between stages of lactations are also shown outside of the diagram.

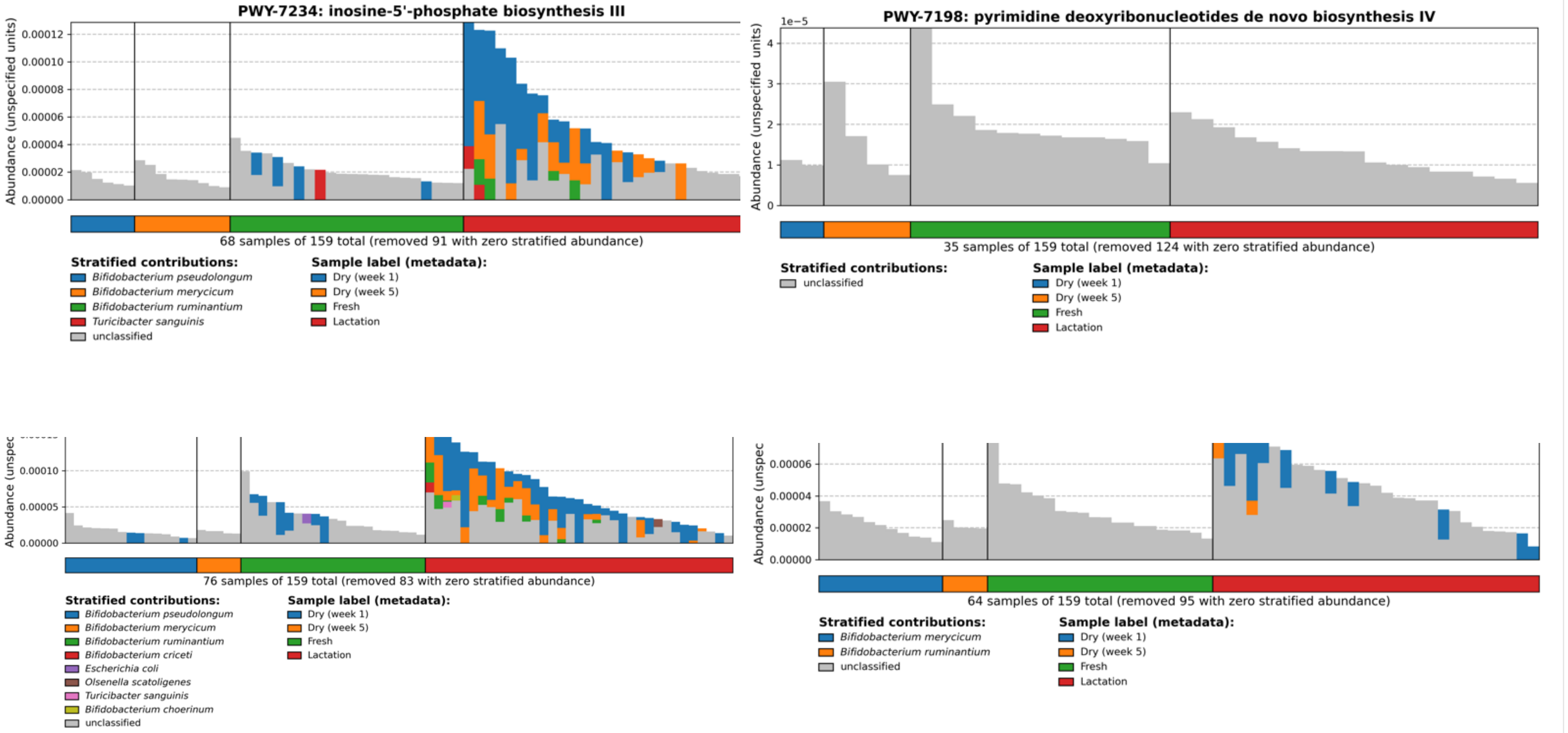

Figure S7. Significantly different cell-division-related pathways between samplings.

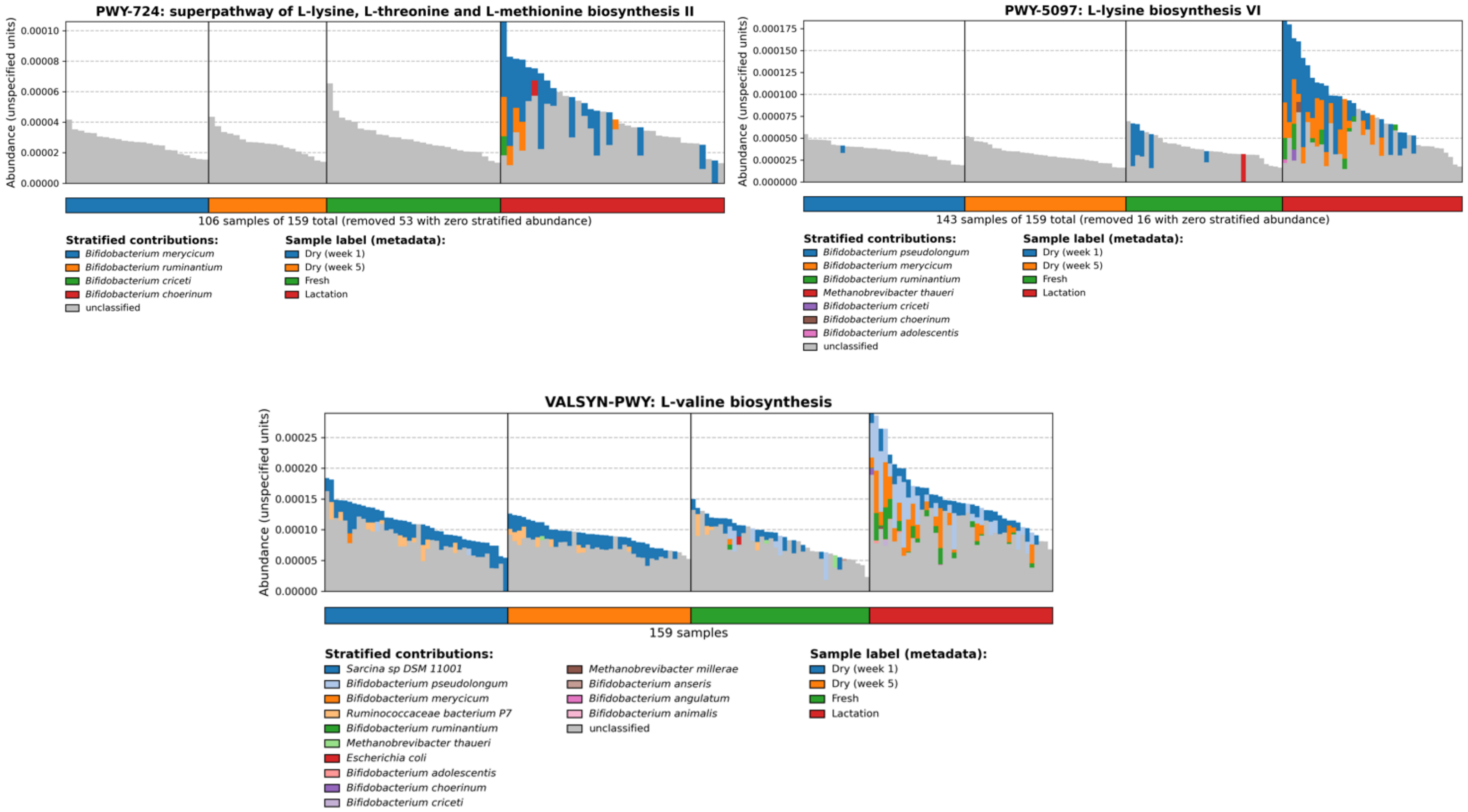

Figure S8. Amino acid biosynthesis pathways were significantly different across the sampling period.

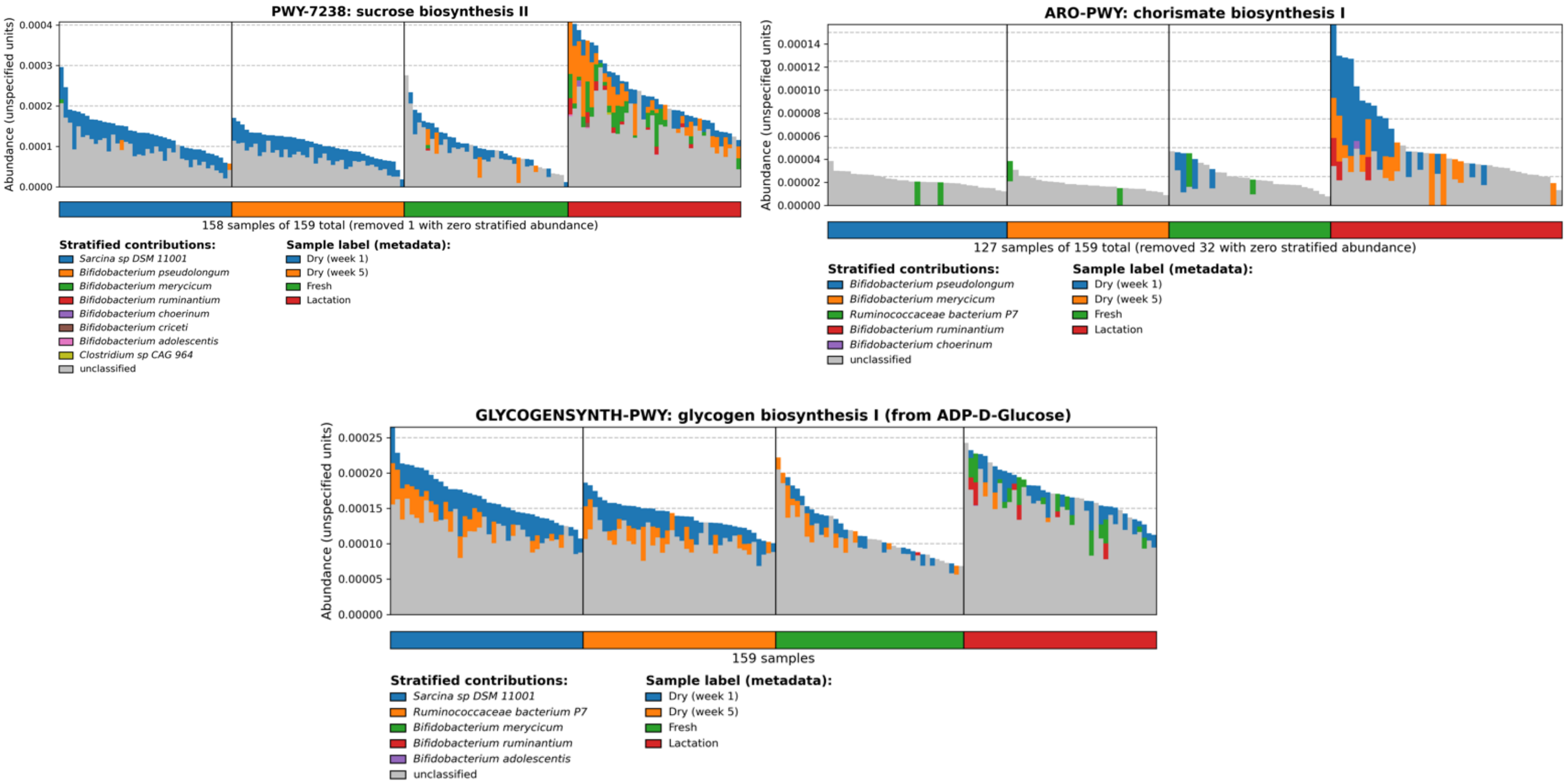

Figure S9. Carbohydrate biosynthesis pathways were significantly different between samplings.

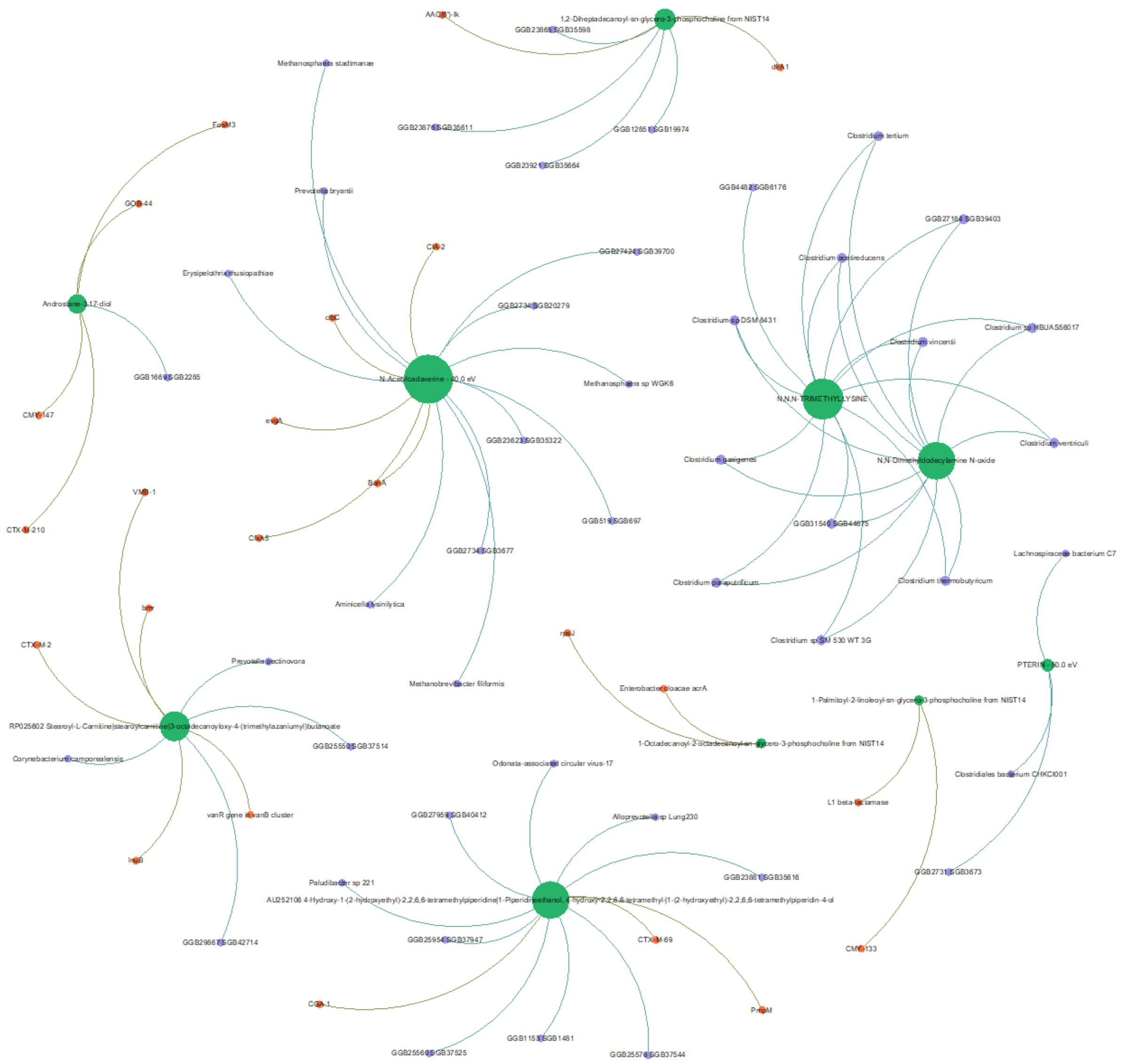

Figure S10. Correlation network among polar metabolites and microbiome. Node size correspond to the degree of centrality, while colors correspond to metabolites (green), ARGs (orange) and taxa (purple).

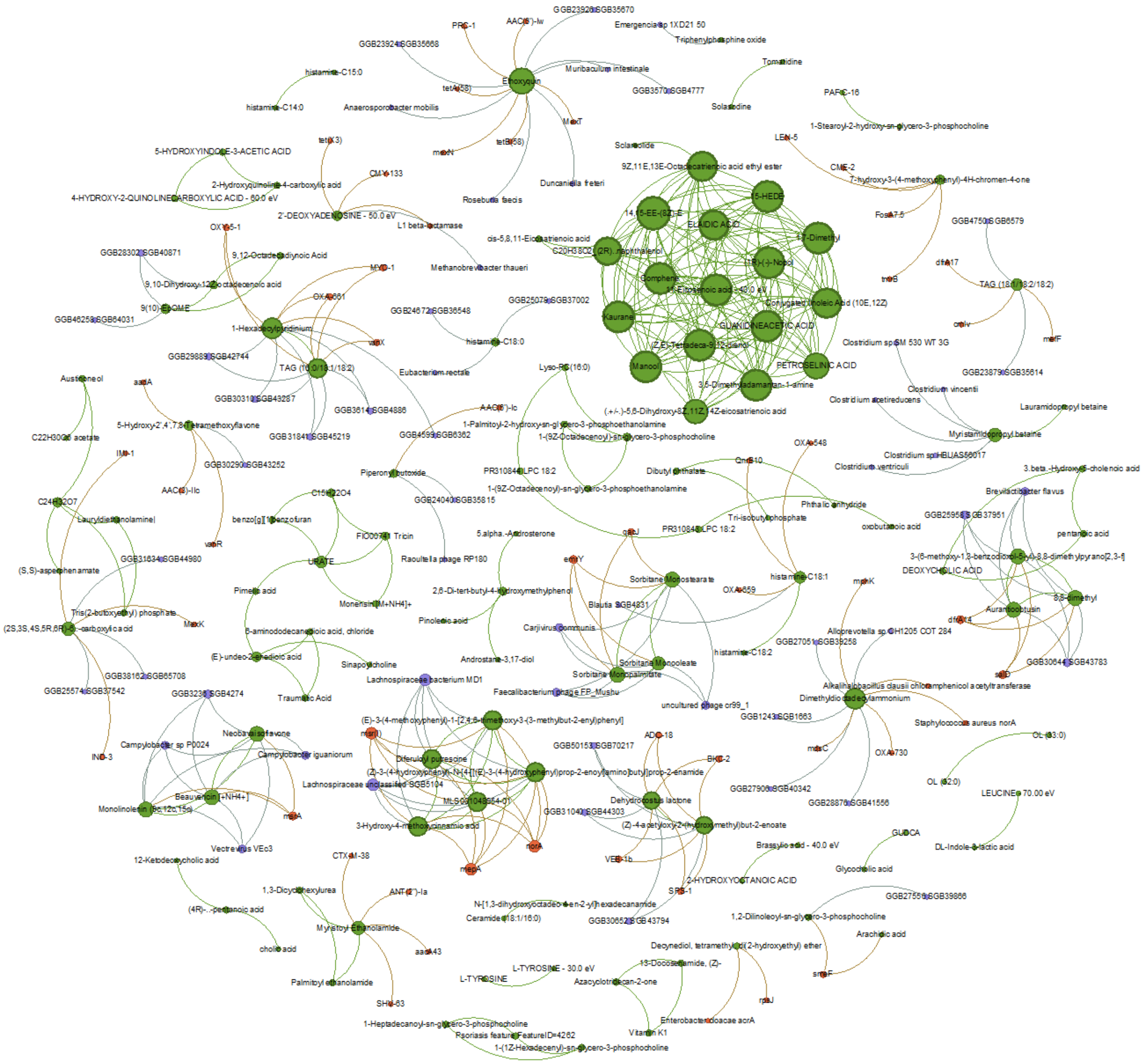

Figure S11. Correlation network between nonpolar metabolites and microbiome. Node size correspond to the degree of centrality, while colors correspond to metabolites (green), ARGs (orange) and taxa (purple).

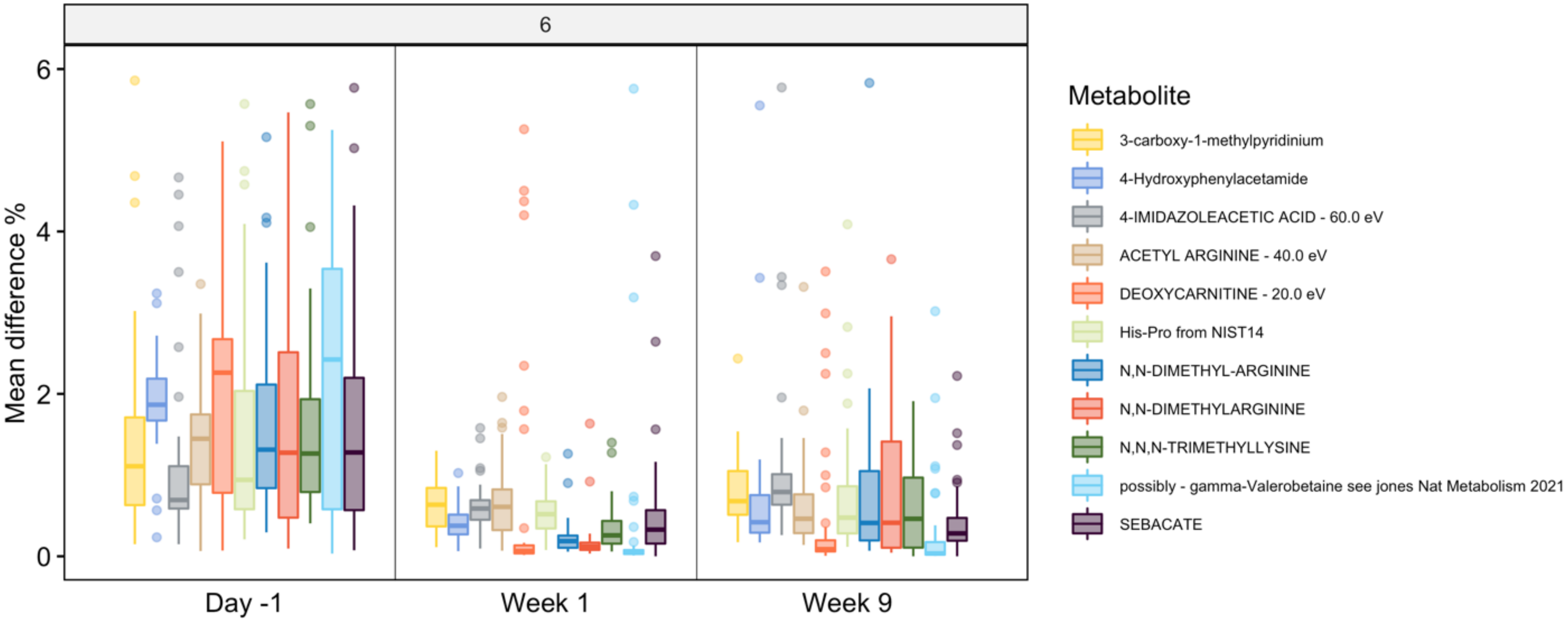

Figure S12. Group of polar metabolites that were significantly higher in each sampling.

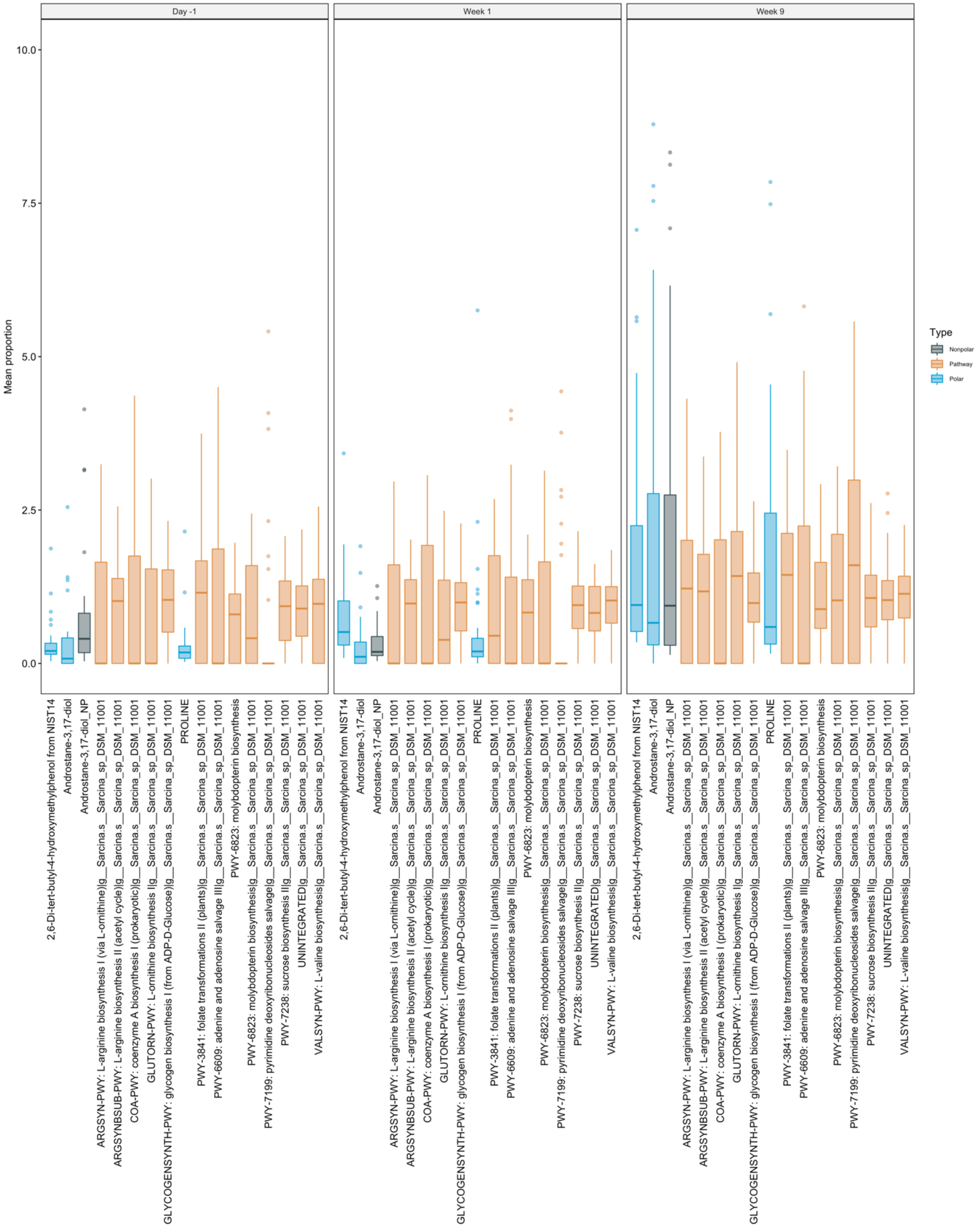

Figure S13. Cluster of microbial pathways and polar and nonpolar metabolites that were significantly higher in fresh cows.
